# Recognition of brain activities via graph-based long short-term memory-convolutional neural network

**DOI:** 10.3389/fnins.2025.1546559

**Published:** 2025-03-24

**Authors:** Yanling Yang, Helong Zhao, Zezhou Hao, Cheng Shi, Liang Zhou, Xufeng Yao

**Affiliations:** ^1^School of Health Science and Engineering, University of Shanghai for Science and Technology, Shanghai, China; ^2^College of Medical Imaging, Jiading District Central Hospital Affiliated Shanghai University of Medicine and Health Sciences, Shanghai, China

**Keywords:** magnetoencephalography (MEG), motor imagery (MI), cognitive imagery (CI), graph convolutional network (GCN), long short-term memory (LSTM), spatial convolution

## Abstract

**Introduction:**

Human brain activities are always difficult to recognize due to its diversity and susceptibility to disturbance. With its unique capability of measuring brain activities, magnetoencephalography (MEG), as a high temporal and spatial resolution neuroimaging technique, has been used to identify multi-task brain activities. Accurately and robustly classifying motor imagery (MI) and cognitive imagery (CI) from MEG signals is a significant challenge in the field of brain-computer interface (BCI).

**Methods:**

In this study, a graph-based long short-term memory-convolutional neural network (GLCNet) is proposed to classify the brain activities in MI and CI tasks. It was characterized by implementing three modules of graph convolutional network (GCN), spatial convolution and long short-term memory (LSTM) to effectively extract time-frequency-spatial features simultaneously. For performance evaluation, our method was compared with six benchmark algorithms of FBCSP, FBCNet, EEGNet, DeepConvNets, Shallow ConvNet and MEGNet on two public datasets of MEG-BCI and BCI competition IV dataset 3.

**Results:**

The results demonstrated that the proposed GLCNet outperformed other models with the average accuracies of 78.65% and 65.8% for two classification and four classification on the MEG-BCI dataset, respectively.

**Discussion:**

It was concluded that the GLCNet enhanced the model’s adaptability in handling individual variability with robust performance. This would contribute to the exploration of brain activates in neuroscience.

## 1 Introduction

Magnetoencephalography (MEG) is a non-invasive neuroimaging technique that records dynamic spatiotemporal brain patterns with millisecond resolution by measuring the magnetic fields produced by neuronal activity (Yang et al., 2024). Revealing the developmental patterns of motor cognition requires brain magnetic measurement technology with high temporal and spatial resolution, and MEG is the ideal tool for detecting the complex and multifaceted development of cognitive abilities (Gross, 2019). Compared to other neuroimaging modalities, MEG offers substantial advantages, including high temporal resolution, the capacity for direct measurement of neuronal activity, real-time data acquisition, and precise localization of dynamic neural processes (Hämäläinen et al., 2020; Mellinger et al., 2007). Recent studies have demonstrated MEG’s effectiveness in classifying specific motor imagery (MI) and cognitive imagery (CI). For instance, MEG has been applied to classify upper limb movements (Hesse et al., 2007), recognize multiple gestures (Bu et al., 2023), and assess kinematic parameters (Kim et al., 2023), offering new possibilities for applications in fields such as neurorehabilitation and human-computer interaction (Halme and Parkkonen, 2018). These findings underscore MEG’s potential for enhanced classifying of brain activity and its applicability in diverse research and clinical settings.

Brain activities such as MI (Groß et al., 2002; Jerbi et al., 2007), CI (Leeuwis et al., 2021), motor execution (ME) (Solodkin et al., 2004), and emotional processing (Dumas et al., 2011) are key areas for understanding neural dynamics. Among them, MI holds promise in rehabilitation medicine, sports training, and neuroscience research (Chholak et al., 2019). MI involves mentally rehearsing movements without physically executing them (Phadikar et al., 2023), which is especially valuable for identifying movement intentions in patients with motor impairments and enables the implementation of closed-loop neurofeedback systems to support rehabilitation (Mane et al., 2020). CI is particularly significant for preventing cognitive decline and treating cognitive impairments (Hooda and Kumar, 2020). It involves activating specific brain regions through mental task simulation, generating interpretable neural patterns essential for both research and clinical practice. Till now, MEG decoding continues to face challenges in explaining complex brain activities and enhancing classification efficiency. While some progresses have been made in specific tasks, such as feature extraction from high-dimensional signals, understanding of brain activities, and accuracy improvement.

Classification of MEG signals in different task states can reveal brain region interactions and information processing processes (Nara et al., 2023; Lotte et al., 2018). However, challenges such as noise, high inter-trial variance, and limited training data, coupled with high intra-class variability of MI and CI, make MEG classification studies more difficult (Lotte et al., 2018; Roy et al., 2019). Previous studies on MI classification can be divided into two categories: classical machine learning (ML) and deep learning (DL) methods. Classical ML methods have mainly depended on manually crafted features derived from neurophysiological signals; however, these methods are frequently hindered by their time-consuming nature, dependency on individual subjects, and limited ability to extract effective features (Lotte et al., 2018). Handcrafted features can result in suboptimal classification performance due to the inherent limitations of individual expertise and experience. In contrast, DL enables the training of end-to-end models that directly map raw signals to categories, automatically extracting high-level features necessary for classification and thereby reducing the reliance on handcrafted features (Ju and Guan, 2022; Altaheri et al., 2023; Al-Saegh et al., 2021). DL models still struggle to meet the stringent demands for high accuracy and robust generalization required for emerging clinical applications (Sakhavi et al., 2018).

Among the classical ML methods, common spatial pattern (CSP) is a powerful method for constructing optimal spatial filters (Ramoser et al., 2000; Ang et al., 2008). As a result, several extended CSP variants have been developed, including filter bank CSP (FBCSP) (Ang et al., 2008) and discriminative filter bank CSP (DFBCSP) (Thomas et al., 2009). For feature classification, various classical classifiers are often employed, such as linear discriminant analysis (LDA) (Fisher, 1936), support vector machines (SVMs), and random forest (RF) (Breiman, 2001), are commonly used to classify MI tasks (Lu et al., 2010; Ang et al., 2012; Zheng et al., 2018). Rathee et al., (2021) used the FBCSP method for feature extraction, with low inter-session comparison accuracy and an average classification rate of about 69.35% for specific tasks (hand vs. word generation). Youssofzadeh et al. (2023) applied SVM classification to beta power decrements, achieving accuracy rates of 74% for MI tasks (Hand vs. Feet) and 68% for CI tasks (Word vs. Sub). Tang et al. (2024) employed SVM with a radial basis function (RBF) kernel to categorize features derived from Riemannian geometry, achieving an average accuracy of up to 80.47% in two-class cross-session MEG data tasks. Inspired by the success of EEGNet (Lawhern et al., 2018; Sarma et al., 2023) proposed MEGNet, a compact deep neural network model designed to improve single-trial decoding. MEGNet effectively captures essential spatiotemporal features and improves classification accuracy, which results showed that MEGNet outperforms traditional feature extraction methods, achieving a consistent mean accuracy of 64.76% for two classification. Bu et al. (2023) developed the MEG-RPSnet model, a convolutional neural network for decoding hand gestures from MEG signals, and achieving an average accuracy of 85.56% for Rock-Paper-Scissors gestures. Wang et al. (2024) performed unilateral movement decoding of the upper and lower limbs using MEG signals. Their study demonstrated that source-level analysis had achieved an averaged accuracy of 96.97% in classifying lower limb tasks, significantly outperforming sensor-level analysis.

MEG and electroencephalography (EEG) are non-invasive brain activity recording techniques with shared electromagnetic foundations, capturing electrical and magnetic fields from synchronized cortical neurons and providing complementary insights into brain dynamics (Hämäläinen et al., 1993; Cohen, 1972). Although there are limited studies applying DL to MEG classification, the data characteristics of MEG and EEG are similar and both reflect neural activities. Several DL-based EEG classification models have been proposed (Lawhern et al., 2018; Schirrmeister et al., 2017; Mane et al., 2021), thus providing a reference for MEG research based on DL.

Recent studies have investigated the potential of DL methods, with particular focus on convolutional neural networks (CNNs) (Mane et al., 2020) and recurrent neural networks (RNNs) (Luo et al., 2018), as promising solutions for EEG signal classification. Lawhern et al. (2018) introduced the EEGNet model, which uses a two-step convolutional sequence to learn spatial filters for each time filter, effectively extracting spatial features of specific frequencies. The results demonstrated that the model achieves four-class classification accuracy of 73.15%. Schirrmeister et al. (2017) designed the DeepConvNet, a deep convolutional network inspired by successful architectures in computer vision, to address the task of EEG decoding. The results demonstrated that DeepConvNet achieves four-class classification accuracy of 72.22%. Schirrmeister et al. (2017) developed Shallow ConvNet, drawing inspiration from the FBCSP approach, to capture the temporal dynamics of band power fluctuations within trials (Sakhavi et al., 2015). Mane et al. (2021) proposed FBCNet, which utilizes multi-view data representation and spatial filtering techniques to extract spectral-spatial features with discriminative power. The model achieved four-class classification accuracy of 76.20%. Furthermore, the integration of CNN with graph theory led to the development of the graph convolutional network (GCN), which incorporates the functional topological relationships of brain networks (Defferrard et al., 2016; Song et al., 2018; Wang et al., 2019). Hou et al. (2022) proposed GCNs-Net, designed to classify four-class MI tasks by leveraging the functional topological relationships between electrodes. This method demonstrated reliable convergence for both individual and group-level predictions, achieving an average accuracy of 93.06% on the PhysioNet dataset. RNNs and long short-term memory (LSTM) networks have also been proposed to capture temporal features for EEG classification (Yang et al., 2020; Hu et al., 2020; Wang et al., 2018). While GCN and LSTM have achieved high prediction accuracy, few researchers have explored this approach in the context of MEG classification.

To address the limitation of low accuracy in MEG multi-task classification, we developed a graph-based long short-term memory convolutional neural network (GLCNet) for the classification of MEG signals. Our model is characterized by the implementation of three main modules: the GCN module, which efficiently extracts spatial features; the spatial convolution module, performing spatial convolutions across all channels at each time point; and the LSTM module, capturing the temporal dynamics of the MEG signal, respectively. Finally, the spatial features extracted from the GCN and the spatiotemporal features captured from both the spatial convolution and LSTM modules are fed into a fully connected layer for precise classification results. Therefore, the time-frequency-spatial features of MEG signals are fully exploited by combining modules. The contributions are summarized as follows:

(1) An improved GLCNet model is proposed to efficiently classify MEG signals. This model integrates three key modules: GCN, spatial convolution, and LSTM. It effectively captures highly discriminative and robust features.
(2) An enhanced cross-entropy loss function will be added to focus the model on difficult samples, accelerating their correction and promoting faster convergence.
(3) Experiment results show that the GLCNet outperforms benchmark algorithms on the MEG-BCI datasets.

## 2 Materials and methods

### 2.1 MEG datasets

We evaluated the classification accuracy on two datasets, namely the MEG-BCI datasets (Rathee et al., 2021) and the BCI competition IV dataset 3.^1^

#### 2.1.1 MEG-BCI datasets

The dataset utilized in this study is an open-source MEG-BCI datasets designed for MI and CI tasks (Rathee et al., 2021). The dataset consists of 17 participants, of whom 14 are male (82.35%) and 3 are female (17.64%), with the median age being 28 years.

MEG data were acquired using a 306-channel whole-head neuromagnetometer system (Elekta Neuromag TRIUX; MEGIN Oy, Helsinki, Finland), consisting of 204 planar gradiometers and 102 magnetometers. The experiments were conducted in a shielded room, and the raw data were acquired with on-line filtering from 0.01 to 300 Hz at a sampling rate of 1,000 Hz. Head position tracking started and continued 20 s after the start of the experiment (Rathee et al., 2021).

**Figure 1:**
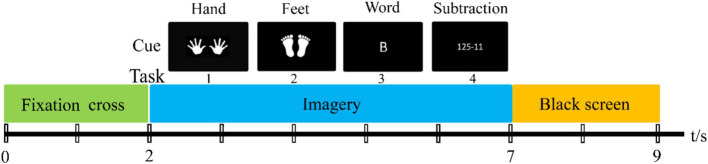
Timing diagram of MEG-BCI paradigm.

The experimental paradigm included four mental imagery tasks: both hands movements (Hand), both feet movements (Feet), mathematical subtraction (Sub) and word generation (Word). The timing diagram of the MEG-BCI paradigm is shown in Figure 1. Each trial starts with a 2-s rest period, followed by a 5-s imagery task period (Rathee et al., 2021). Each subject’s two-session experiment was conducted on different days, and each session contained 200 trials (4 classes × 50trials).

#### 2.1.2 BCI competition IV dataset 3

BCI Competition IV dataset 3 includes MEG signals of two subjects, S1 and S2. The MEG changes during wrist movements in four directions with the right hand were measured by 10 MEG channels located above the motor areas. Those signals were filtered with a 0.5–100 Hz band-pass filter, and resampled at 400 Hz. There were 40 trials per target direction, resulting in a total of 160 labeled trials for each subject. The category labels of the four movement directions (right, forward, left, backward) were set to 1, 2, 3, and 4, respectively. The data format of MEG signal was represented as: number of experiments × number of time sampling points × number of channels. Here, the training data are 160 × 400 × 10 and 160 × 400 × 10, and the test data are 74 × 400 × 10 and 73 × 400 × 10 for S1 and S2, respectively.

#### 2.1.3 MEG data preprocessing

For MEG-BCI datasets, we selected 16 subjects (excluding participant with ID ‘sub-2’, whose information is incomplete), resulting in a total of 6,400 samples (16 subjects × 2 sessions × 200 trials), MEG data from the 204 gradiometer sensors were utilized. Next, we performed preprocessing on the MEG data using MNE-Python (Gramfort et al., 2013). Bad channel detection, jump artifact correction, and head movement compensation were performed using signal space projection (SSP) and Maxwell filtering techniques. Following this, MEG data were downsampled to 500 Hz, signal space separation (SSS) removes external noise sources (such as power lines, wireless devices, etc.). The data were baseline corrected within the time window of −200 ms to 0 ms. Each trial spanned 5,000 ms, from 2,000 ms to 7,000 ms, followed by 0.5–40 Hz bandpass filtering and removal of artifacts using independent component analysis (ICA). For BCI competition IV dataset 3, we used the commonly used time window segmentation of [0, 4] s for data analysis.

### 2.2 Proposed GLCNet

**Figure 2:**
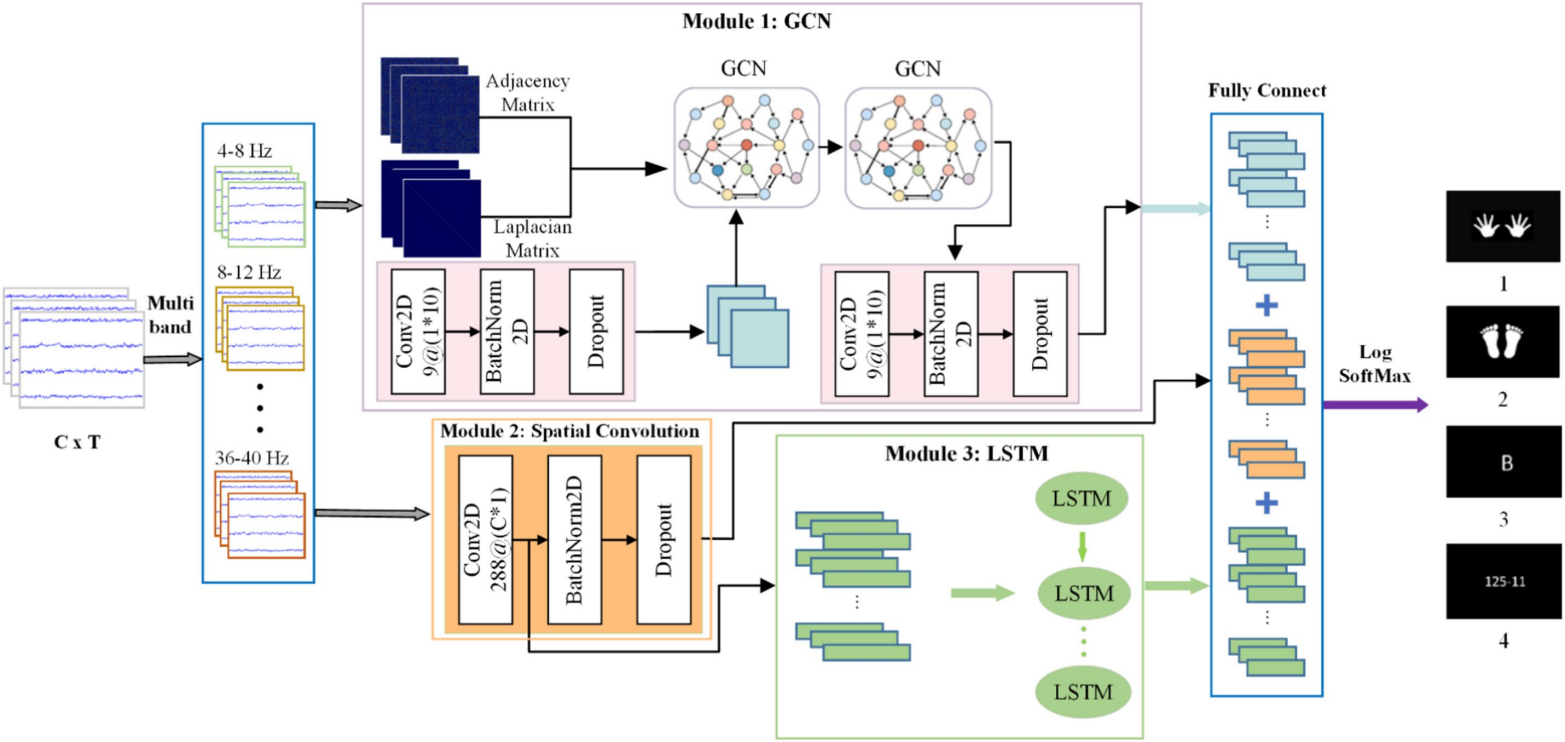
The framework of the proposed GLCNet.

As shown in Figure 2, the proposed GLCNet consists of five vital parts: multi-band layers, GCN module, spatial convolution module, LSTM module, and fully connected layer. The multi-band layer divides MEG signals into multiple frequency sub-bands for analyzing brain activities within specific frequency ranges; the GCN module obtains functional topological relationships between MEG channels relying on their relational properties; the spatial convolution module extracts spatial dependencies among neighboring channels; the LSTM module captures long-term temporal features by analyzing global correlations across time points. Finally, the fully connected layer integrates acquired features to yield predicted results.

The raw MEG data is first input into the multi-band layer to obtain multiple frequency sub-bands, which are then sequentially passed through the GCN module and spatial convolution module. The GCN outputs highly generalized MEG spatial features that capture graph correlations. Next, the spatial representations obtained from the spatial convolution module are input into the LSTM module, which outputs features with enhanced temporal correlations. Finally, the spatiotemporal features extracted by the GCN and spatial convolution-LSTM modules are integrated and forwarded to a fully connected layer to produce the classification results.

Table 1 contains the main parameters and layer configurations of each module of GLCNet. Here, C, T denote the number of channels and time points, respectively. F represents the number of filters in the convolutional layers, and K indicates the size of the convolution kernel. ELU and ReLU activation functions are applied to the convolution and classification layers, respectively. S represents stride; the probability parameter P in dropout refers to the probability of a single neuron being discarded; A represents the adjacency matrix, while D denotes the degree matrix. Nc represents category.

**Table 1:** Detailed framework and parameters of GLCNet.

Module	Layer name	Hyper parameters	Output shape	Number of parameters	Activation
Multi-band	Input	[4:4:40]	(9,C,T)		
Module 1: GCN	Conv-2D	*K* = (1,10) *F* = 9	(9,C,T)	8,109	ReLU
	BatchNorm2D	*F* = 9		18	ELU
	MaxPool2D	*K* = (1,10)	(9,C,T/10)		
		*S* = (1,10)			
	Dropout	*p* = 0.25			
	GCN	$A\in R^{204\times 204}$ $D_{i,j}=\underset{j}{\sum }A_{ij}$	(9,C,64)	15,424	ReLU
	GCN	$A\in R^{204\times 204}$ $D_{i,j}=\underset{j}{\sum }A_{ij}$	(9,C,5)	325	ReLU
	Conv-2D	*K* = (204,1) *F* = 288	(288,1,5)	529,056	ReLU
	BatchNorm2D	*F* = 288			ELU
	Dropout	*p* = 0.25			
Module 2: Spatial convolution	Conv-2D	*K* = (204,1) *F* = 288	(288,1,T)	59,040	ReLU
	BatchNorm2D	*F* = 288		576	
	Average time	*T* = 500	(288,T/500,1)		
Module 3: LSTM	LSTM	*S* = 1	(288,T/500,1)	44,002	
Fully connect	Conv-2D	*K* = (288*T/500)	( $N_{c}$ ,1,1)	5,764	ReLU
	LogSoftMax		( $N_{c}$ ,1,1)		

#### 2.2.1 Multi-band layers

This study applies a multi-band layer to decompose MEG signals into frequency sub-bands, generating multi-view representations that facilitate the analysis of frequency-specific brain activity. GLCNet employs a multi-view representation of the MEG data, *x,* generated by applying a filter bank with narrow-band temporal filters to the raw MEG signal. The filter bank F, comprising non-overlapping frequency bands (4–8, 8–12, …, 36–40 Hz) with 4 Hz bandwidths, utilizes a Chebyshev Type II filter for the filtering process. The design of this module is based on inspiration from the FBCSP algorithm (Ang et al., 2008), providing sufficient frequency resolution to capture spatial patterns within the signal’s frequency components. Frequency bands based on typical MEG signal distributions were chosen to capture distinct characteristics of brain activity. This approach significantly improves the recognition of spatial patterns across different frequency bands for providing richer neural activity details.

#### 2.2.2 GCN module

Due to the non-Euclidean spatial arrangement of MEG channels, graph-based data structures are highly effective for representing brain connectivity. We propose utilizing a GCN to capture the spatial relationships among MEG channels. By incorporating advancements in graph theory, the GCN efficiently captures spatial features in MEG signals by learning the underlying relationships between nodes. These spatial dependencies can be efficiently represented and visualized as graphs, with each channel corresponding to a node and edges indicating the connections between channels. GCN performs convolution operations on graph-structured data in non-Euclidean space, where the graph captures the spatial relationships, $G=\left(V,E\right)$ consists of nodes V and edges E. The adjacency matrix $A\in R^{Ns\times Ns}$ describes the connections between different nodes (Zhao et al., 2019). The MEG signal, comprising both time-domain and channel features, contains spatially distributed information across channels. Thus, applying graph convolution to the channel dimension is critical for enhancing model performance (Du et al., 2022). The convolutional propagation rule in GCN for updating node features at each layer is defined in Equation 1.


(1) $$H^{\left(l+1\right)}=\sigma \left({\tilde{D}}^{-\frac{1}{2}}\tilde{A}{\tilde{D}}^{\frac{1}{2}}H^{\left(l\right)}W^{l}\right)$$

where $\tilde{A}$ is the adjacency matrix of the graph *G* plus the identity matrix, ${\tilde{D\;}}^{\left(1\right)}$ is the degree matrix, $H^{\left(l\right)}$ is the activation unit matrix of the *l*-th layer, $W^{\left(l\right)}$ is the parameter matrix of each layer, and *σ* represents the nonlinear activation function (Du et al., 2022). In this study, we use the phase lag index (PLI) matrix to replace the manually set adjacency matrix in the GCN (Piqueira, 2011).

**Figure 3:**
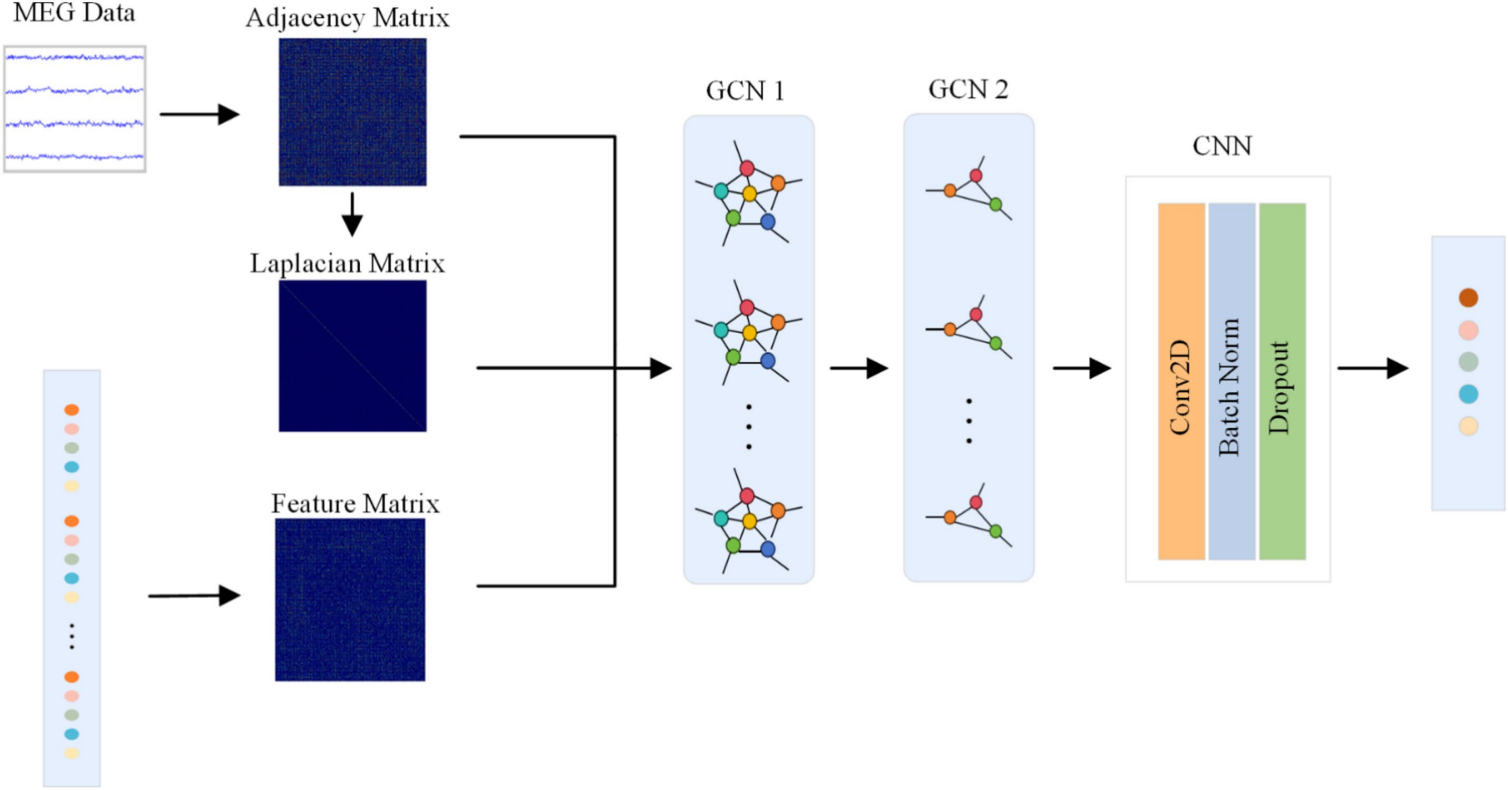
The structure of the GCN module.

The structure of the GCN module is illustrated in Figure 3. Multi band MEG input into GCN is represented as graph $G=\left(V,E\right)$ , where the nodes correspond to the set of MEG |V|=Ns and E the edges with $|E|=N_{S}\times \left(N_{S}-1\right)/2$ . E is represented as an adjacency matrix $A\in R^{Ns\times Ns}$ with $A_{j,k}=1-d\left(S_{j,}S_{k}\right)$ , where *d* is the min-max normalized geodesic distance. *L* is represented as a Laplacian matrix. The CNN output, Z, represents the characteristic feature vectors of G, with the adjacency matrix, Laplacian matrix, and characteristic matrix sequentially introduced to capture the topological structure of the features as a graph. During forward propagation of the network, graph nodes are computed pointwise convolution is used to update node features based on the features of neighboring nodes, edge weights and learnable parameters. The architecture consists of two graph convolutional layers with dimensions (64,5) specified by the network’s design, followed by a CNN layer that aggregates node features into a single embedding vector of length 5.

#### 2.2.3 Spatial convolution module

The spatial convolution module draws inspiration from the spatial convolution block (SCB) layer of FBCNet (Mane et al., 2021). It applies convolutional filters across the spatial dimensions of MEG data to capture dependencies and relationships between channel locations. This module enhances the extraction of spatial features by capturing interactions between neighboring channels, thereby improving the identification of patterns and structures in the brain’s spatial activity domain. The spatial convolution module utilizes 2D convolution kernels to extract channel-specific spatial features (Peng et al., 2021). Following the 2D convolution, BatchNorm2D and dropout are applied to reduce overfitting.

#### 2.2.4 LSTM module

**Figure 4:**
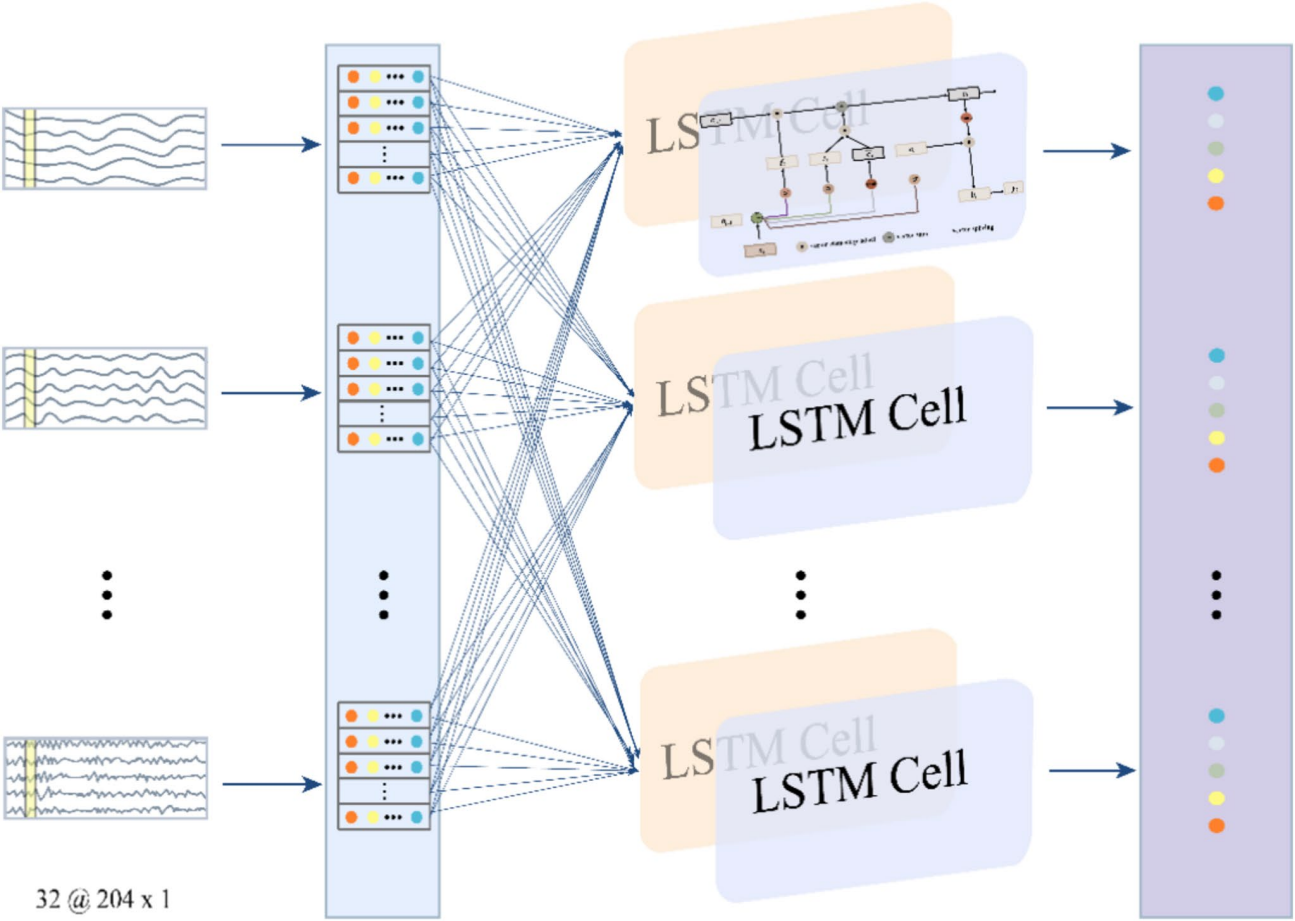
The structure of the LSTM module.

The LSTM, a variant of RNN, incorporates storage units and a gating mechanism to replace standard hidden layer updates, effectively addressing the vanishing gradient issue in traditional RNNs. It excels in processing multivariate time series data, such as MEG signals, by capturing both short- and long-term dependencies to extract temporal features (Yu et al., 2019). The related formulas can be expressed in Equations 2–6, where w and b are the weights and biases of different layers in the memory, σ is sigmoid activation function, i_t_,f_t_,o_t_,c_t_,h_t_ are input gate, forget gate, out gate, cell state and hidden state (He et al., 2022; Saichand, 2021). Figure 4 illustrates the structure of the LSTM module.


(2) $$i_{t}=\sigma \left(w_{i}x_{t}+U_{i}h_{t-1}+b_{i}\right)$$

(3) $$f_{t}=\sigma \left(w_{f}x_{t}+U_{f}h_{t-1}+b_{f}\right)$$

(4) $$o_{t}=\sigma \left(w_{o}x_{t}+U_{o}h_{t-1}+b_{o}\right)$$

(5) $$c_{t}=f_{t}⊙c_{t-1}+i_{t}⊙{\tilde{c\;}}_{t}$$

(6) $$h_{t}=o_{t}⊙\tanh\left(c_{t}\right)$$

In MEG signal processing, LSTM segments the signal into multiple time intervals and sequentially processes each segment to update internal states and generate outputs. This approach captures complex temporal features that reflect MEG signal dynamics. The resulting feature matrix from LSTM processing includes a feature vector for each segment, capturing both instantaneous values and temporal evolution. These features enhance classification accuracy and model robustness.

### 2.3 Loss function of enhanced cross-entropy

The loss function plays a crucial role in evaluating a model’s performance by quantifying the difference between predicted and actual outcomes. The cross-entropy (CE) loss function is widely used to measure the divergence between predicted and target probabilities (Ho and Wookey, 2019). The probability distribution *p* represents the true labels, and *q* the predicted outputs. The CE is given by $H\left(p,q\right)$ , with the CE for multi-class classification computed as shown in Equation 7:


(7) $$H\left(p,q\right)=-\underset{x}{\sum }\left(p\left(x\right)\mathrm{logq}\left(x\right)\right)$$

It can be observed that a smaller $H\left(p,q\right)$ indicates a closer alignment between the two probability distributions, which signifies more accurate prediction results. In the MEG dataset, samples exhibit varying characteristics and can be categorized based on the ease with which the model can classify them. Some samples are straightforward for the model to predict, often referred to as “easy samples.” Conversely, others present more challenges and are known as “difficult samples.” While traditional CE yields small losses for easy samples, an abundance of such samples can dominate the overall loss, overshadowing the contributions from more difficult samples. The loss function should prioritize difficult samples, allowing the model to focus on them and improve MI classification accuracy (Ma et al., 2023).

To shift the model’s focus toward challenging samples, the CE formula has been refined, resulting in the introduction of the enhanced cross-entropy (ECE) loss function. The ECE introduces weights into the original formula, with smaller weights for easier samples and larger weights for more difficult samples, which is expressed as Equation 8.


(8) $$H\left(p,q\right)=-\underset{x}{\sum }{\left(1-\sqrt{q\left(x\right)}\right)}^{2}\left(p\left(x\right)\mathrm{logq}\left(x\right)\right)$$

In this formula, the larger the value of $q\left(x\right)$ , the easier the sample is to classify. Consequently, a smaller value of ${\left(1-\sqrt{q\left(x\right)}\right)}^{2}$ indicates that this easy sample contributes less to the overall loss, while difficult samples, with larger ${\left(1-\sqrt{q\left(x\right)}\right)}^{2}$ values, contribute more significantly. By introducing the square root, the loss function is designed so that, for correctly predicted samples, $H\left(p,q\right)$ is smaller than the traditional CE, whereas for incorrectly predicted samples, $H\left(p,q\right)$ is larger. This adjustment directs the loss function to prioritize incorrectly predicted samples, accelerating the model’s error correction and promoting faster convergence.

### 2.4 Experimental setup

The experiment consisted of two-class and four-class classifications. For MEG-BCI dataset, the two-class experiments included Hand vs. Feet, Hand vs. Word, Hand vs. Subtraction, Feet vs. Word, Feet vs. Subtraction, and Word vs. Subtraction. The four-class classification among Hand, Feet, Word, and Subtraction tasks was conducted. For BCI competition IV dataset 3, the two-class classification of 1 vs. 2, 1 vs. 3, 1 vs. 4, 2 vs. 3, 2 vs. 4, 3 vs. 4 and the four-class classification for 1, 2, 3, and 4 tasks were performed.

To evaluate the performance of GLCNet on the public MEG-BCI datasets, the performance of GLCNet was compared with six state-of-the-art benchmark algorithms of FBCSP (Ang et al., 2008), FBCNet (Mane et al., 2021), EEGNet (Lawhern et al., 2018), Deep ConvNets (Schirrmeister et al., 2017), Shallow ConvNet (Schirrmeister et al., 2017), and MEGNet (Sarma et al., 2023). In order to validate the effectiveness of GLCNet, we performed a technical validation of the four-classification model in terms of model training, ablation studies, feature visualization, comparison of ECE and CE accuracy, and statistical analysis. Among them, the ablation experiments are divided into three groups: (a) removal of the GCN module (without module 1); (b) removal of the spatial convolution module (without module 2); and (c) removal of the LSTM module (without module 3).

Two stage training method is employed where the dataset is divided separately into training set and validation set in the first stage. The model was trained using the training set, with early stopping criteria based on the validation accuracy. Training was terminated if no improvement in validation converges within 50 epochs. Setting the maximum number of iterations to 100 ensures sufficient training duration. Once the stopping criterion was met, the model parameters corresponding to the best validation accuracy were reinstated. In the second stage the validation set is added to the training set and the validation set loss is monitored and training stops when it is lower than the first stage.

The training method for GLCNet is shown in Algorithm 1. In the first stage, the input is processed through modules 1–3 to compute three feature sets (F1-F3), where F3 uses only the spatial convolution layer from module 2. These features are then fused by summation, and the resulting combined feature set is passed to a fully connected layer, which applies the ECE loss function to output the loss value of the fused feature set. In the second phase, the validation set is integrated with the training set, and the same training procedure as in the first phase is followed.

**ALGORITHM 1:**
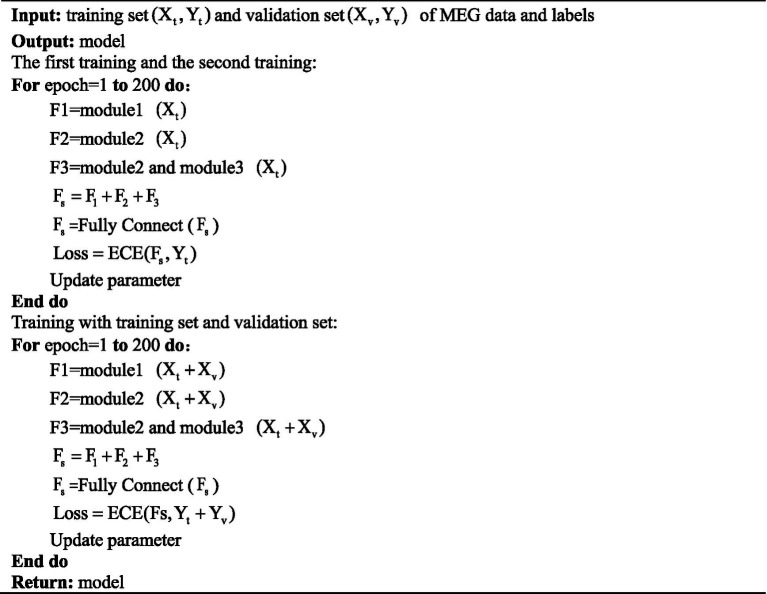
Training detail of proposed GLCNet.

The proposed GLCNet is implemented with PyTorch 1.13.1 on four NVIDIA GeForce RTX 3090 GPUs platform. The training process was configured with a batch size of 32, a learning rate of 0.001, and the ECE loss function. The Adam optimizer and ReLU activation function were employed, while Dropout with a rate of 0.25 was used to mitigate overfitting. These settings of hyperparameters were referenced from FBCNet (Mane et al., 2021) and Deep ConvNets (Schirrmeister et al., 2017). The running code is available on GitHub.^2^

Three metrics were used for performance evaluation, which are expressed in Equations 9–11.


(9) $$\mathrm{Accuracy}=\frac{TP+TN}{TP+FN+FP+TN}$$

(10) $$F1=2\times \frac{\mathrm{Recall}\times \mathrm{Precision}}{\mathrm{Recall}+\mathrm{Precision}}$$

(11) $$K=\frac{Acc-p_{e}}{1-p_{e}}$$

TP , TN , FP , FN represent true positives, true negatives, false positives, and false negatives, respectively. Recall refers to the proportion of actual positive samples that are correctly identified as positive. Precision and recall are often considered opposing metrics. To account for both metrics, the F1-score was introduced. $P_{e}$ represents the accuracy of classification under random conditions. Additionally, the kappa value serves as a measure of agreement, providing an assessment of classification consistency.

## 3 Results

### 3.1 Results of model training

**Figure 5:**
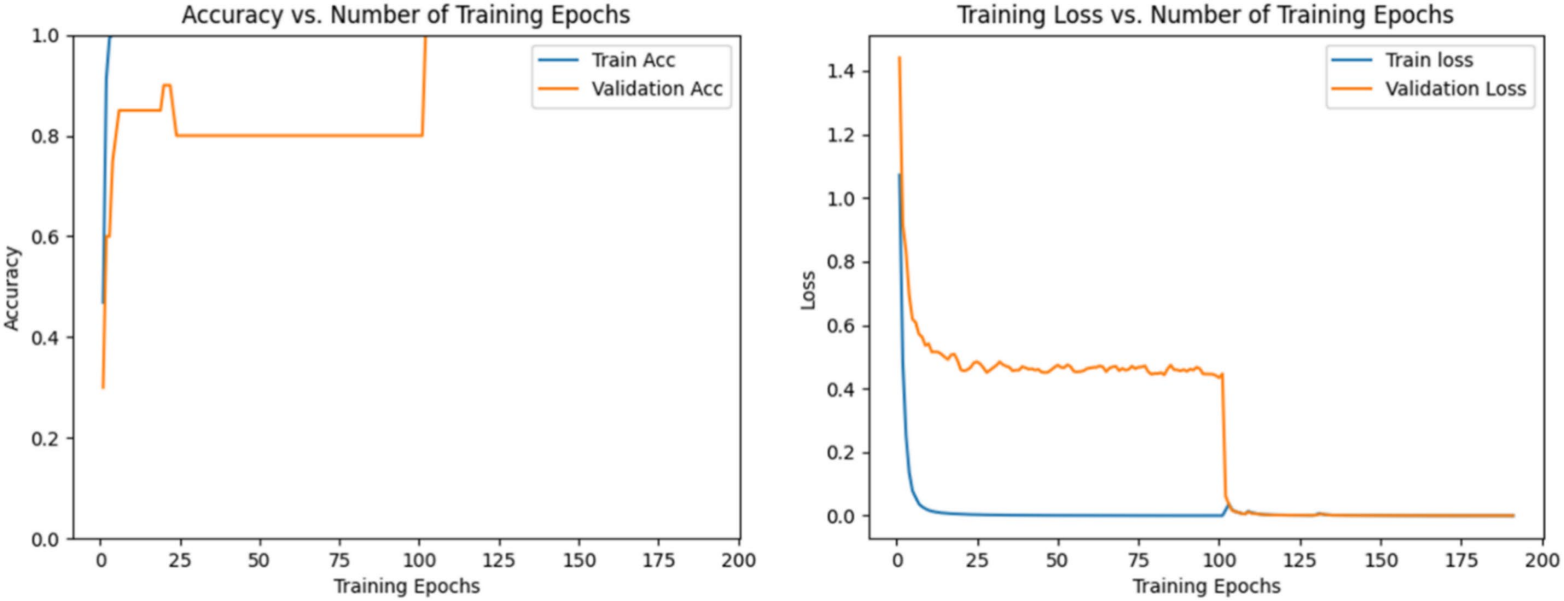
Accuracy and loss of GLCNet in the training and validation sets during training.

The process involves two stages. In the first stage, prior to the 100th epoch, the validation set remains completely separate from the training set. During this stage, accuracy and loss converge quickly, with validation accuracy stabilizing around 80%, indicating that GLCNet is well-trained. In the second stage, following the 100th epoch, in order to make full use of all the training samples, the verification set is added to the training set as a new training set. Consequently, the validation data are incorporated into both training and validation processes, leading to validation accuracy approaching 100%. Additionally, the loss values for both the training and validation sets show a close alignment. The accuracy and loss trends of GLCNet during the training process are shown in Figure 5.

### 3.2 Results of ablation experiments

As shown in Table 2, removing any module results in a decrease in accuracy. Module 1 had the most significant impact, with recognition accuracy dropping by 9.00%. This module is primarily responsible for extracting spatial features; a 3.10% decrease after removing module 2, which extracts spatial convolution features; and a 5.70% decrease after removing module 3, responsible for temporal feature extraction. The positive impact of module 1 and 3 on model performance are evident, as the classification accuracy of GLCNet significantly declines when either module is excluded.

**Table 2:** The accuracy comparison in the ablation study on MEG-BCI dataset.

Dataset	w/o module 1		w/o module 2		w/o module 3		GLCNet	
	Acc (%)	Kappa	Acc (%)	Kappa	Acc (%)	Kappa	Acc (%)	Kappa
MEG-BCI	56.8	0.39	62.7	0.51	60.1	0.51	65.8	0.55

### 3.3 Feature visualization

**Figure 6:**
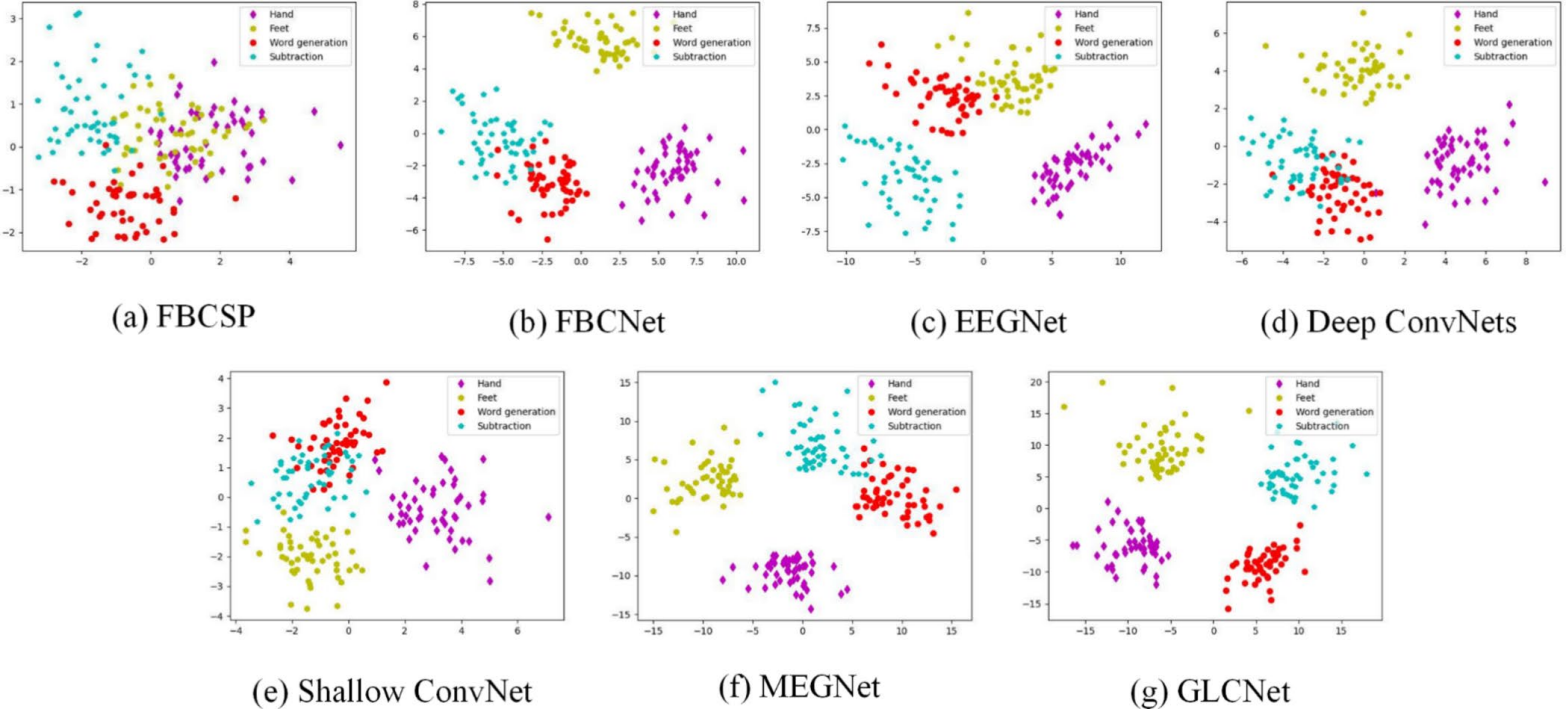
Visualization of t-SNE for sub 11 of MEG-BCI dataset. The four colors of dots represent Hand, Feet, Word Generation, and Subtraction.

The t-distributed stochastic neighbor embedding (t-SNE) (Van Der Maaten and Hinton, 2008) is a popular visualization method, which is employed to generate a two-dimensional embedding of the learned MEG features. Figure 6 shows the t-SNE visualization (sub11) of the feature distributions produced by the different models for the four tasks: hand, feet, word generation and subtraction. Compared to the other models, GLCNet has a higher discrimination with the least overlap between classes.

### 3.4 Effectiveness of ECE loss function

**Figure 7:**
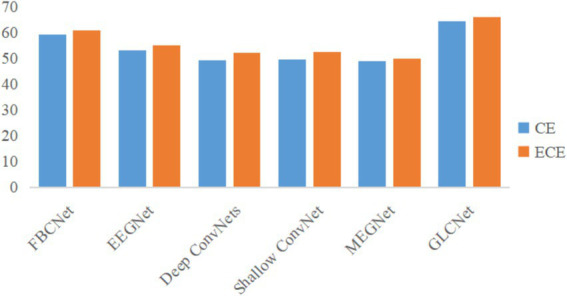
Comparison of loss functions of CE and ECE for GLCNet and other models.

To validate the effectiveness of the proposed ECE loss function in classifying challenging samples in MEG data and enhancing classification accuracy, it is essential to demonstrate its practical impact on improving the model’s training performance. As illustrated in Figure 7, we compared the average accuracy of five benchmark algorithms and GLCNet using both the CE and ECE loss functions. The results show an approximately 2% improvement in the average classification accuracy for each model when using ECE. This indicates that the proposed ECE loss function is more effective in enhancing the classification accuracy, particularly for challenging samples.

**Figure 8:**
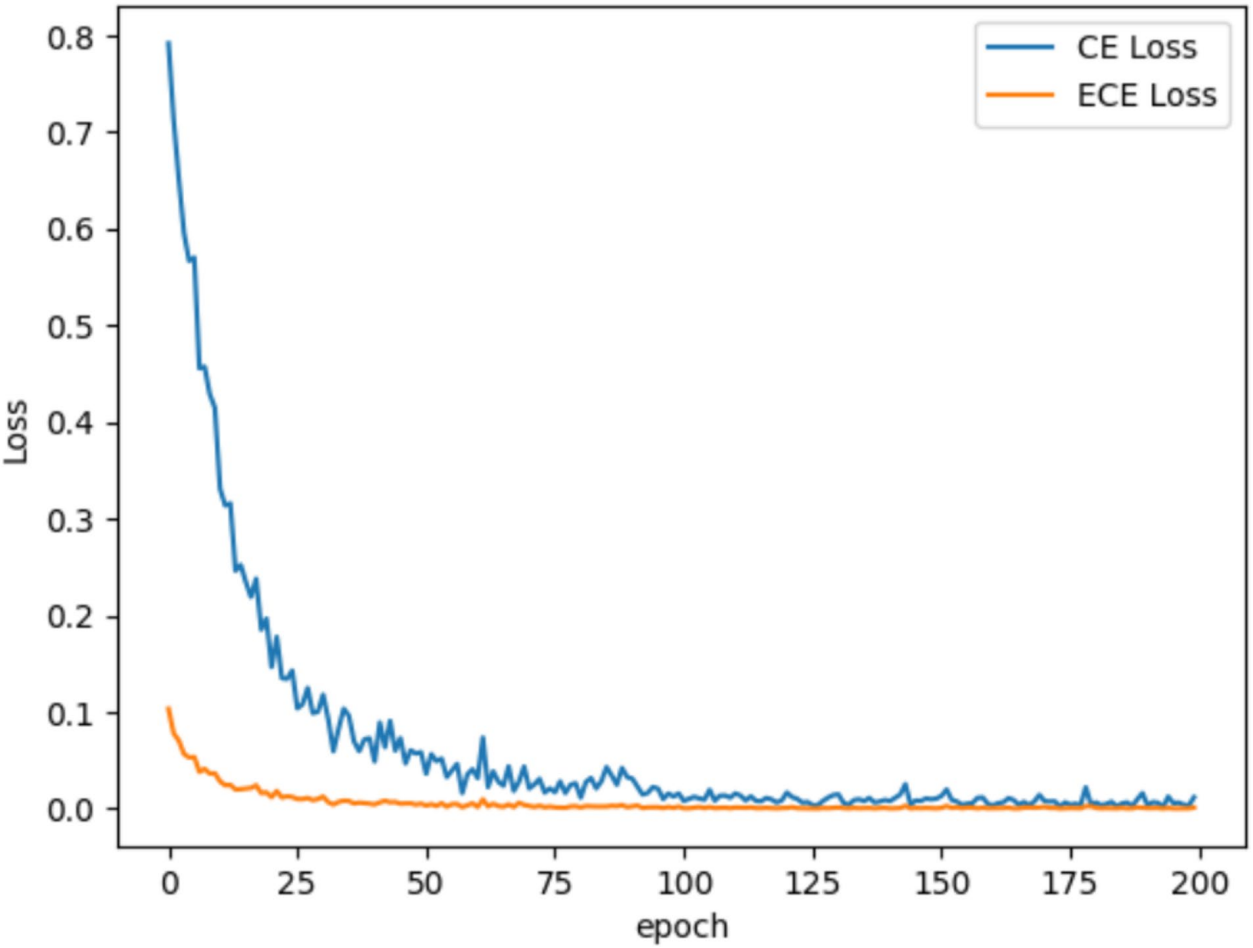
Comparison of convergence curves for CE and ECE on the sub 11 for MEG-BCI dataset.

Figure 8 compares the convergence curves of CE Loss and ECE Loss for the GLCNet model on sub 11 of MEG-BCI dataset. It is clear that the ECE Loss (orange curve) decreases more quickly than CE Loss (blue curve) along with epochs. This meant that the lower ECE Loss reflected more accurate predicted probabilities and exhibited stronger capabilities.

### 3.5 MEG classification on MEG-BCI

#### 3.5.1 Results of two-class classification

The average comparison results of the six two-class classification tasks are presented in Table 3. Overall, for the classifications of Hand vs. Feet, Hand vs. Word, Hand vs. Subtraction, Feet vs. Subtraction, and Word vs. Subtraction, the GLCNet model achieves superior mean accuracies of 70.8, 82.7, 81.3, 81.7, and 78.9%, respectively, outperforming the other six algorithms. In Supplementary Table S1 (Hand vs. Feet), FBCNet, EEGNet and MEGNet achieve mean accuracies about 65%, while FBCSP, DeepConvNets, and ShallowConvNet achieve mean accuracies around 60%. In Supplementary Table S2 (Hand vs. Word), the mean accuracies for FBCSP, FBCNet, EEGNet, DeepConvNets, ShallowConvNet, and MEGNet were 66.3, 79.6, 72.4, 70.0, 70.2, and 72.0%, respectively. In Supplementary Table S3 (Hand vs. Subtraction), MEGNet accuracy around 77.0%, both DeepConvNets and ShallowConvNet recorded accuracies of 73%. While FBCNet and EEGNet showed steady performance, FBCSP consistently yielded lower classification results. In Supplementary Table S4 (Feet vs. Word), GLCNet achieves an average accuracy of 76.5%, slightly below FBCNet’s 77.9%. FBCSP, EEGNet, DeepConvNet and MEGNet achieved 61.4, 73.3, 68.5, and 71.63%, respectively. In Supplementary Table S5 (Feet vs. Subtraction), FBCSP recorded the lowest accuracy at 69.8%, FBCNet maintained approximately 77%, both EEGNet and ShallowNet reached 73%, MEGNet recorded the lowest accuracy at 50.3%. In Supplementary Table S6 (Word vs. Subtraction), FBCSP again recorded the lowest accuracy at 59.1%, FBCNet remained around 77%, and EEGNet, DeepConvNets, ShallowNet and MEGNet each scored approximately 70%.

**Table 3:** Comparison of accuracy, kappa and F1-score for two-class classification for proposed model and other models.

TASK	FBCSP			FBCNet			EEGNet			Deep ConvNets			Shallow ConvNet			MEGNet			GLCNet		
	Acc (%)	K	F1	Acc (%)	K	F1	Acc (%)	K	F1	Acc (%)	K	F1	Acc (%)	K	F1	Acc (%)	K	F1	Acc (%)	K	F1
H-F	59.1	0.20	0.50	64.9	0.29	0.62	65.5	0.28	0.63	59.6	0.28	0.63	58.6	0.17	0.56	65.0	0.30	0.64	**70.8**	**0.38**	**0.68**
H-W	66.3	0.33	0.60	79.6	0.55	0.76	72.4	0.41	0.70	70.0	0.41	0.70	70.2	0.38	0.67	72.0	0.44	0.72	**82.7**	**0.66**	**0.83**
H-S	65.5	0.31	0.60	79.0	0.55	0.75	80.1	0.57	0.78	74.4	0.57	0.78	72.1	0.45	0.70	77.0	0.54	0.77	**81.3**	**0.63**	**0.81**
F-W	61.4	0.20	0.60	**77.9**	**0.53**	**0.75**	73.3	0.43	0.70	68.5	0.43	0.70	70.8	0.38	0.68	72.0	0.42	0.70	76.5	0.52	0.74
F-S	69.8	0.40	0.70	77.0	0.51	0.73	74.0	0.50	0.74	73.0	0.50	0.74	72.6	0.43	0.70	50.0	0.07	0.32	**81.7**	**0.62**	**0.81**
W-S	59.1	0.18	0.50	76.4	0.52	0.75	72.6	0.40	0.69	70.0	0.40	0.69	66.3	0.31	0.63	71.0	0.43	0.70	**78.9**	**0.58**	**0.78**

The bold values indicate the highest values for each metric within each task.

**Figure 9:**
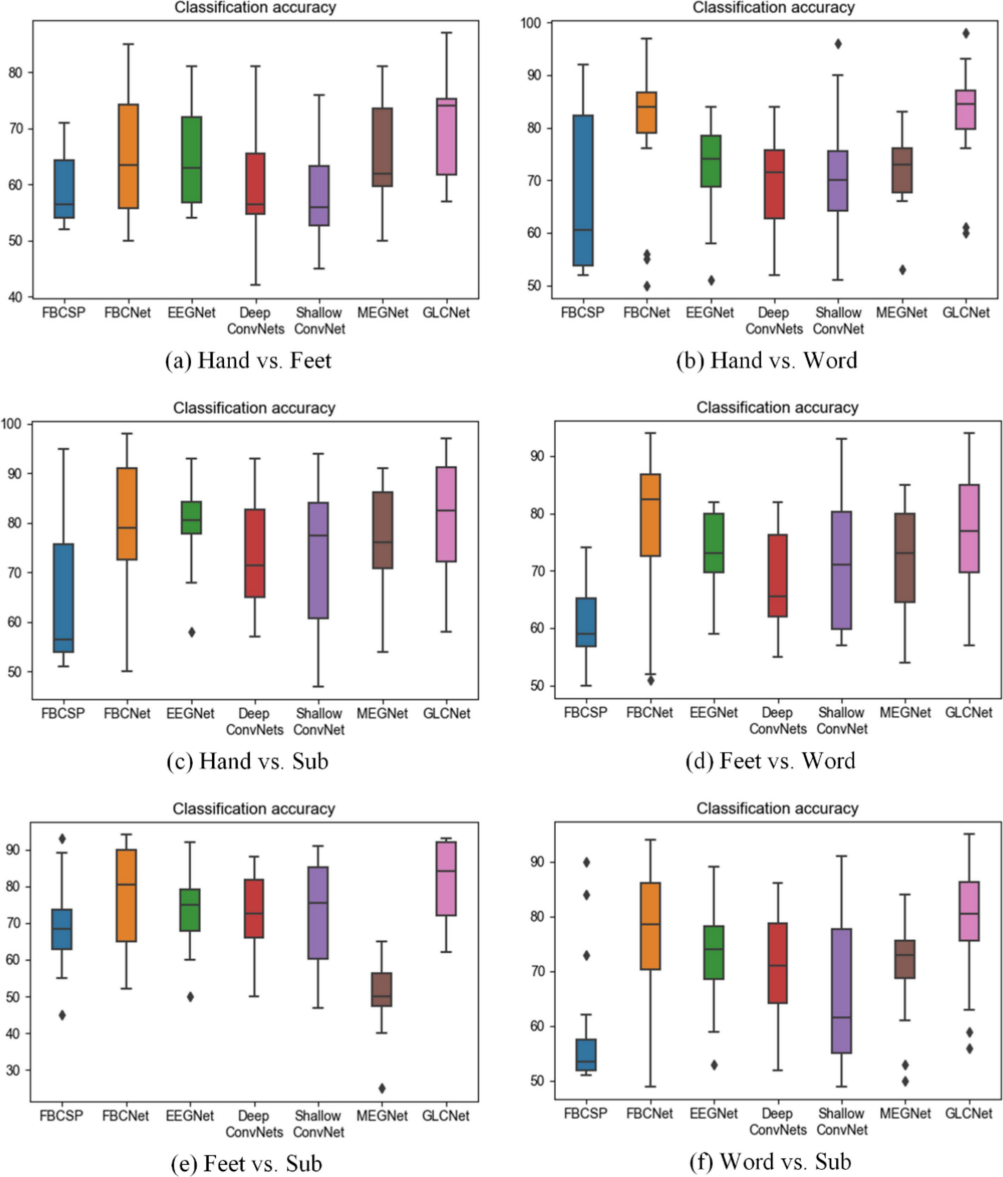
Comparison of two-class classification accuracy for GLCNet and other models. Black dots indicate outliers.

In conclusion, Figure 9 summarizes the results, showing that the proposed GLCNet achieved a maximum accuracy of 82.7% in two-class classification, demonstrating superior performance over the other six algorithms. Both FBCSP, EEGNet and MEGNet exhibited stable performance, while FBCNet maintained consistent accuracy across classifications. DeepConvNets and ShallowConvNet showed moderate classification performance, with FBCSP being the least effective among the algorithms tested.

#### 3.5.2 Results of four-class classification

**Figure 10:**
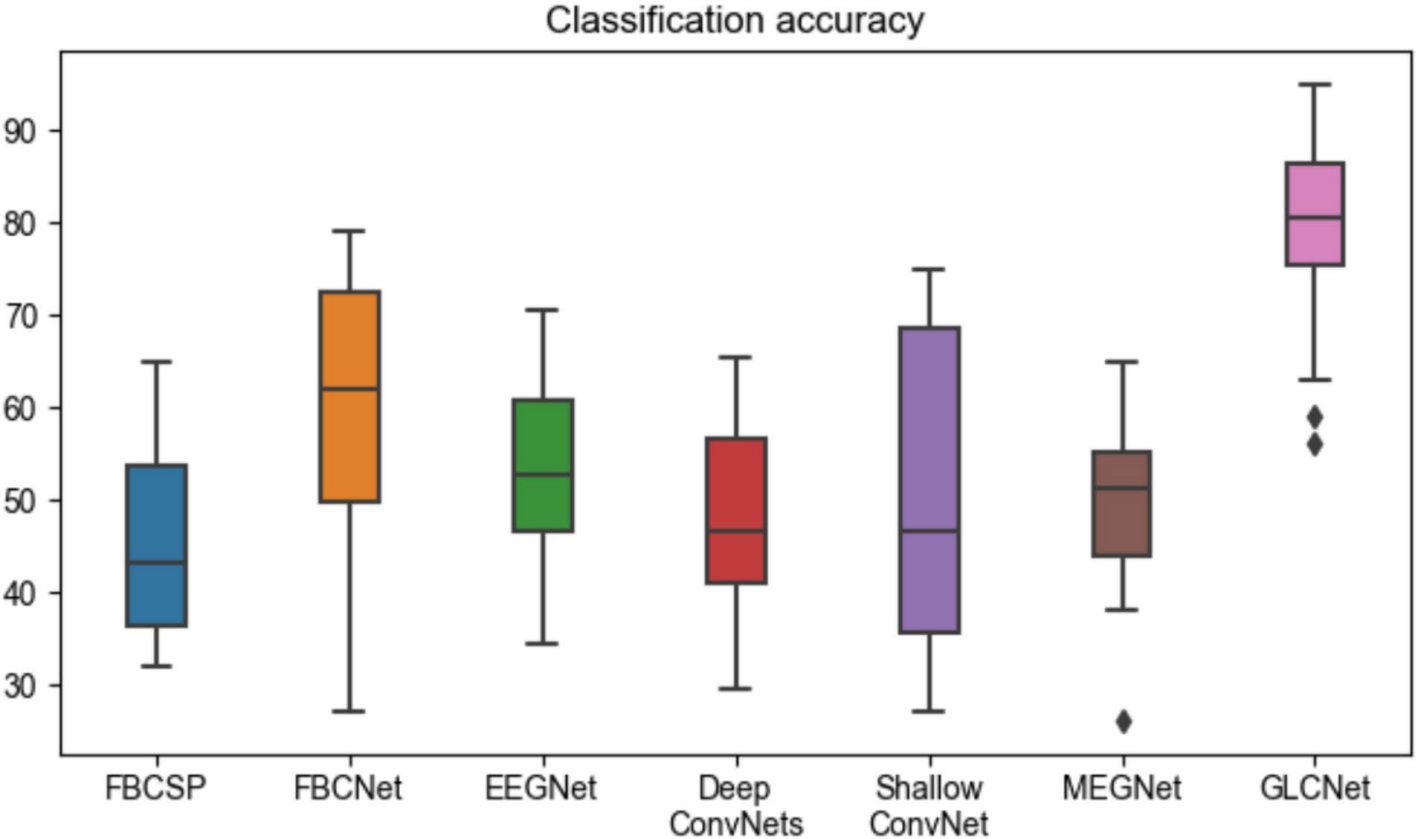
Comparison of four-class classification accuracy for GLCNet and other models. Black dots indicate outliers.

Table 4 and Figure 10 present the complete four-class classification results on the MEG-BCI dataset using the proposed GLCNet and other benchmark algorithms. Overall, GLCNet surpasses the benchmarks in average classification accuracy, achieving an average accuracy of 65.8%, a mean kappa value of 0.55, and a mean F1-score of 0.65. This represents improvements of 20.7, 6.7, 12.8, 16.7, 16.1, and 15.9% over FBCSP, FBCNet, EEGNet, DeepConvNets, ShallowConvNet, and MEGNet, respectively. Furthermore, the kappa value of 0.55, the highest among all methods, highlights the effectiveness of the proposed approach.

**Table 4:** Comparison of accuracy, kappa and F1-score for four-class classification for proposed model and other models.

Sub.	FBCSP			FBCNet			EEGNet			Deep ConvNets			Shallow ConvNet			MEGNet			GLCNet		
	Acc (%)	K	F1	Acc (%)	K	F1	Acc (%)	K	F1	Acc (%)	K	F1	Acc (%)	K	F1	Acc (%)	K	F1	Acc (%)	K	F1
1	44.0	0.25	0.38	51.0	0.31	0.49	57.0	0.31	0.57	41.0	0.15	0.39	31.5	0.12	0.29	47.0	0.29.	0.47	63.5	0.44	0.63
3	56.0	0.41	0.50	76.0	0.73	0.76	50.0	0.31	0.50	63.5	0.40	0.64	69.5	0.54	0.70	53.0	0.37	0.52	77.5	0.76	0.77
4	32.0	0.09	0.28	27.0	0.02	0.25	34.5	0.09	0.34	29.5	0.07	0.28	27.0	0.07	0.19	26.0	0.01	0.23	45.5	0.33	0.44
6	32.5	0.10	0.29	74.0	0.67	0.74	64.5	0.51	0.64	65.5	0.51	0.65	60.0	0.39	0.57	64.5	0.53	0.64	72.0	0.61	0.72
7	40.0	0.20	0.35	49.5	0.16	0.48	49.0	0.28	0.47	46.5	0.19	0.46	29.0	0.09	0.31	52.0	0.36	0.50	61.5	0.47	0.60
9	55.5	0.41	0.51	50.0	0.36	0.48	51.0	0.27	0.51	53.5	0.38	0.53	28.5	0.06	0.22	40.5	0.21	0.40	75.5	0.75	0.75
11	65.0	0.53	0.64	65.0	0.55	0.65	54.5	0.42	0.49	46.5	0.25	0.44	69.0	0.51	0.68	52.5	0.37	0.50	73.5	0.64	0.73
12	36.5	0.15	0.32	44.5	0.25	0.43	34.5	0.15	0.34	37.0	0.04	0.34	45.5	0.21	0.44	38.0	0.17	0.37	59.5	0.39	0.58
13	43.0	0.24	0.39	63.5	0.48	0.63	47.5	0.35	0.47	46.5	0.21	0.46	48.0	0.25	0.48	50.5	0.34	0.50	58.5	0.57	0.58
14	37.0	0.16	0.32	54.0	0.38	0.53	43.5	0.25	0.43	36.5	0.21	0.36	45.5	0.29	0.44	44.5	0.26	0.44	63.0	0.43	0.62
15	43.5	0.25	0.43	74.5	0.63	0.74	64.5	0.49	0.64	44.5	0.26	0.43	70.0	0.43	0.70	60.0	0.47	0.60	80.0	0.74	0.80
16	36.0	0.15	0.31	65.0	0.03	0.65	57.0	0.34	0.55	55.0	0.39	0.55	43.0	0.22	0.44	48.0	0.31	0.48	64.0	0.49	0.64
17	36.0	0.15	0.34	39.5	0.17	0.35	41.5	0.23	0.42	40.5	0.19	0.40	37.0	0.13	0.33	42.5	0.23	0.43	48.0	0.31	0.46
18	53.0	0.37	0.51	72.0	0.65	0.71	70.5	0.62	0.70	64.0	0.48	0.63	75.0	0.62	0.75	65.0	0.53	0.65	73.5	0.72	0.73
19	48.0	0.31	0.45	60.5	0.45	0.60	59.5	0.39	0.60	59.5	0.40	0.60	47.5	0.27	0.46	53.5	0.38	0.53	62.0	0.49	0.61
20	63.5	0.51	0.61	79.0	0.69	0.79	68.5	0.54	0.69	55.5	0.37	0.54	68.5	0.51	0.68	61.0	0.48	0.61	75.5	0.71	0.76
Avg.	45.1	0.27	0.42	59.1	0.41	0.58	53.0	0.35	0.52	49.1	0.28	0.48	49.7	0.29	0.48	49.9	0.32	0.49	**65.8**	**0.55**	**0.65**

The bold values indicate the highest values for each metric across all models for each subject.

Table 5 presents the statistical significance of GLCNet compared to other models. With the exception of FBCNet, the classification results of the remaining four benchmark algorithms differ significantly from GLCNet (*p* < 0.001), indicating that GLCNet performs significantly better than these benchmark algorithms.

**Table 5:** ANOVA statistical analysis results of GLCNet and other models.

	FBCSP	FBCNet	EEGNet	Deep ConvNets	Shallow ConvNet	MEGNet
ref	Ang et al. (2008)	Mane et al. (2021)	Lawhern et al. (2018)	Schirrmeister et al. (2017)	Schirrmeister et al. (2017)	Sarma et al. (2023)
*p*-values	0.001	0.144	0.002	0.001	0.003	0.001

### 3.6 MEG classification on BCI competition IV dataset 3

This study compares six benchmark models for two-class and four-class classification on the BCICIV-3 dataset. In Table 6, the GLCNet obtained the highest accuracies, particularly in the tasks 1 vs. 2, 2 vs. 3, 2 vs. 4, 3 vs. 4, and 1 vs. 2 vs. 3 vs. 4, achieving the accuracies of 76.5, 75.1, 78.1, 77.5, and 59.9%, respectively; the MEGNet also performed well in the tasks of 1 vs. 3 and 1 vs. 4, with the accuracies of 82.9 and 78.1%, respectively. Meanwhile, it was indicated that the FBCNet and FBCSP gave worst results.

**Table 6:** Accuracies (%) of benchmark models for the BCICIV-3 dataset.

Tasks	FBCSP	FBCNet	EEGNet	Deep ConvNets	Shallow ConvNet	MEGNet	GLCNet
1 vs. 2	45.9	49.1	72.9	71.3	61.1	74.3	**76.5**
1 vs. 3	62.6	63.6	75.7	76.3	65.1	**82.9**	79.7
1 vs. 4	31.5	39.3	72.1	69.8	60.1	**78.1**	75.9
2 vs. 3	47.8	48.3	68.8	66.1	60.5	71.4	**75.1**
2 vs. 4	53.6	54.3	75.1	67.8	60.7	73.9	**78.1**
3 vs. 4	40.8	41.3	75.3	70.6	54.8	76.6	**77.5**
4 class	26.7	28.9	53.4	51.1	45.5	55.7	**59.9**

The bold values indicate the maximum accuracy of the models for each task.

## 4 Discussion

This study introduced GLCNet, a novel end-to-end CNN architecture designed for MEG signal classification, combining GCN for spatial feature extraction and LSTM for temporal continuity. Through evaluation on MEG-BCI datasets and BCI competition IV dataset 3, GLCNet outperforms state-of-the-art benchmark algorithms in both classification accuracy and model robustness. Beyond classification accuracy, GLCNet demonstrated strong performance in model training, ablation experiments, feature visualization, comparison of ECE and CE accuracy, and ANOVA analysis. This comprehensive performance highlights GLCNet’s robustness and versatility in MEG signal classification and related analyses. GLCNet serving as a powerful tool for MEG signal classification and advancing its applications in intelligent diagnostics and neuroscience research.

MEG data features high spatial and temporal resolution, making it an ideal modality for investigating intricate brain activity. While traditional CNNs use spatial convolution layers to extract spatial features, they face limitations in handling complex spatial topologies and long-range dependencies. Accordingly, the six chosen benchmark approaches exhibited several challenges. Especially, the FBCSP (Ang et al., 2008) mainly relied on manually designed features, and limited its generalizability across tasks and subjects; the FBCNet (Mane et al., 2021) and EEGNet (Lawhern et al., 2018) were prone to overfitting when decoding MI tasks; the Shallow ConvNet (Schirrmeister et al., 2017) demonstrated high variability in reflecting task-dependent performance; the Deep ConvNets (Schirrmeister et al., 2017) incorporated complexity and failed to capture subtle patterns; the MEGNet (Sarma et al., 2023) effectively identified prominent features but might overlook details in more complex tasks. To address these challenges, this paper introduces GCN for enhanced spatial feature learning, effectively capturing non-Euclidean structures and inter-channel correlations within MEG signals, thus uncovering comprehensive spatial information. Additionally, integrating LSTM, which captures long-term dependencies and complex temporal patterns, improves the continuity of time-series information and effectively manages temporal signal changes and dynamic features. GLCNet combines topological connectivity and convolutional features to deeply explore channel topological structures in the spatial domain while capturing temporal characteristics and signal variations. The inclusion of multi-band layers allows the model to focus on specific frequency ranges, capturing frequency components and their spatial patterns to maximize information utilization. By integrating above modules in parallel, GLCNet enhances classification performance, leveraging the rich temporal, frequency, and spatial information in MEG signals. This multidimensional approach enables more precise analysis of complex brain activity, improving the accuracy and robustness of neural data interpretation.

In this study, we utilized data from the 204 gradiometer sensors while excluding data from the 102 magnetometers. This was because that the gradiometers typically exhibited a higher signal-to-noise ratio (SNR), which improved their sensitivity in detecting cortical activity. Additionally, the gradiometers could provide superior spatial resolution for precise localization of cortical activity (Rathee et al., 2021). The proposed GLCNet model was tested on MEG-BCI and BCI competition IV dataset 3, achieving the highest classification accuracy in both two-class and four-class tasks, except in the Feet vs. Word classification, where FBCNet outperformed GLCNet by 1.4%, and in the 1 vs. 3 and 1 vs. 4 tasks, where MEGNet outperformed GLCNet by 3.2 and 2.2%, respectively. Compared to benchmark algorithms on the MEG-BCI dataset, GLCNet’s average accuracy improved by 8.65% for two-class classification and 14.6% for four-class classification. As the number of tasks grows, the complexity of classification also increases, resulting in a significant performance decline in comparison algorithms for multi-task classification. This highlights the superior performance of GLCNet. Ablation experiments demonstrated that the highest classification performance was obtained when both GCN and LSTM were employed simultaneously. The t-SNE feature distribution visualization confirmed that the GLCNet module enhanced the model’s recognition capabilities. Furthermore, the loss function analysis underscored the benefits of the proposed ECE in improving classification accuracy, particularly for challenging samples.

Our study has several limitations. First, the training samples is very limited. This may affect the generalizability of our proposed approaches in cases of other datasets or populations. Second, the proposed model faces the challenges of long training times and high computational complexity. Third, only one kind of MEG modality was used in our study. The fusion of MEG with multimodal data, such as EEG and/or fMRI would be hopeful for the potential applications in BCI applications.

There are four aspects in our future studies. At first, more samples should be enrolled and even more types of multi-center datasets should be involved. The detailed parameter analyses of age, gender, and health status of samples should be considered in performance evaluation, too. Besides, more data augmentation techniques, such as temporal transformation and frequency filtering, could be tried to resolve the limitation of data size (He and Wu, 2020; Zhang et al., 2021; Ma et al., 2024).

Secondly, more kinds of lightweight alternatives should be explore to alleviate the problem of high training costs, such as model compression (Buciluǎ et al., 2006), knowledge distillation (Gou et al., 2021), and the substitution of lightweight modules (Lahiri et al., 2020) for model simplification.

Thirdly, the fusion of MEG with other modalities, such as EEG and fMRI, is sure to be a potential application in BCI. The multimodal decoding could help to improve model performance by optimizing signal quality, feature extraction (Li et al., 2023). This would assist a lot in the cognitive assessment of clinical applications in neurorehabilitation and cognitive training (Wang et al., 2024).

Finally, the medical ethics would be strictly obeyed in data acquirement, experimental setup and clinical procedures in our further studies. It is true that the MEG would become an important tool in neuroscience, and even in the field of BCI.

## 5 Conclusion

The proposed GLCNet, an advanced end-to-end CNN network, could extract effectively distinguished features from MEG data, and it was concluded that the GLCNet had demonstrated robust capabilities for the classification of MI and CI tasks. It was hopeful to be used in MEG-BCI systems. Future work will pay more attention to enhance the decoding capabilities and explore the potentials of the fused multimodal model in BCI applications.

## Data Availability

The original contributions presented in the study are included in the article/supplementary material, further inquiries can be directed to the corresponding author.

## Footnotes

^1^ https://www.bbci.de/competition/iv/

^2^ https://github.com/yangyanling880/GLCNet_code

## References

[ref1] Al-Saegh A. Dawwd S. A. Abdul-Jabbar J. M. (2021). Deep learning for motor imagery EEG-based classification: a review. Biomed. Signal Proc. Control 63:102172. doi: 10.1016/j.bspc.2020.102172

[ref2] Altaheri H. Muhammad G. Alsulaiman M. Amin S. U. Altuwaijri G. A. Abdul W.. (2023). Deep learning techniques for classification of electroencephalogram (EEG) motor imagery (MI) signals: a review. Neural Comput. & Applic. 35, 14681–14722. doi: 10.1007/s00521-021-06352-5

[ref3] Ang K. K. Chin Z. Y. Wang C. Guan C. Zhang H. (2012). Filter bank common spatial pattern algorithm on BCI competition IV datasets 2a and 2b. Front. Neurosci. 6:39. doi: 10.3389/fnins.2012.00039 22479236 PMC3314883

[ref4] Ang K. K. Chin Z. Y. Zhang H. Guan C. (2008). Filter bank common spatial pattern (FBCSP) in brain-computer interface. Proceeding of the 2008 IEEE international joint conference on neural networks (IEEE world congress on computational intelligence): IEEE), Hong Kong, China. 2390–2397.

[ref5] Breiman L. (2001). Random forests. Mach. Learn. 45, 5–32. doi: 10.1023/A:1010933404324

[ref6] Bu Y. Harrington D. L. Lee R. R. Shen Q. Angeles-Quinto A. Ji Z.. (2023). Magnetoencephalogram-based brain–computer interface for hand-gesture decoding using deep learning. Cereb. Cortex 33, 8942–8955. doi: 10.1093/cercor/bhad173, PMID: 37183188

[ref7] Buciluǎ C. Caruana R. Niculescu-Mizil A. (2006). “Model compression”, Proceedings of the 12th ACM SIGKDD international conference on knowledge discovery and data mining, *Association for Computing Machinery*. 535–541.

[ref8] Chholak P. Niso G. Maksimenko V. A. Kurkin S. A. Frolov N. S. Pitsik E. N.. (2019). Visual and kinesthetic modes affect motor imagery classification in untrained subjects. Sci. Rep. 9:9838. doi: 10.1038/s41598-019-46310-9, PMID: 31285468 PMC6614413

[ref9] Cohen D. (1972). Magnetoencephalography: detection of the brain's electrical activity with a superconducting magnetometer. Science 175, 664–666. doi: 10.1126/science.175.4022.664, PMID: 5009769

[ref10] Defferrard M. Bresson X. Vandergheynst P. (2016). Convolutional neural networks on graphs with fast localized spectral filtering. Curran Associates Inc. 3844–3852.

[ref11] Du G. Su J. Zhang L. Su K. Wang X. Teng S.. (2022). A multi-dimensional graph convolution network for EEG emotion recognition. IEEE Trans. Instrum. Meas. 71, 1–11. doi: 10.1109/TIM.2022.3204314, PMID: 39573497

[ref12] Dumas T. Attal Y. Dubal S. Jouvent R. George N. (2011). Detection of activity from the amygdala with magnetoencephalography. IRBM 32, 42–47. doi: 10.1016/j.irbm.2010.11.001

[ref13] Fisher R. A. (1936). The use of multiple measurements in taxonomic problems. Ann. Eugenics 7, 179–188. doi: 10.1111/j.1469-1809.1936.tb02137.x

[ref14] Gou J. Yu B. Maybank S. J. Tao D. (2021). Knowledge distillation: a survey. Int. J. Comput. Vis. 129, 1789–1819. doi: 10.1007/s11263-021-01453-z

[ref15] Gramfort A. Luessi M. Larson E. Engemann D. A. Strohmeier D. Brodbeck C.. (2013). MEG and EEG data analysis with MNE-Python. Front. Neuroinform. 7:267. doi: 10.3389/fnins.2013.00267, PMID: 24431986 PMC3872725

[ref16] Gross J. (2019). Magnetoencephalography in cognitive neuroscience: a primer. Neuron 104, 189–204. doi: 10.1016/j.neuron.2019.07.001, PMID: 31647893

[ref17] Groß J. Timmermann L. Kujala J. Dirks M. Schmitz F. Salmelin R.. (2002). The neural basis of intermittent motor control in humans. Proc. Natl. Acad. Sci. 99, 2299–2302. doi: 10.1073/pnas.032682099, PMID: 11854526 PMC122359

[ref18] Halme H.-L. Parkkonen L. (2018). Across-subject offline decoding of motor imagery from MEG and EEG. Sci. Rep. 8:10087. doi: 10.1038/s41598-018-28295-z, PMID: 29973645 PMC6031658

[ref19] Hämäläinen M. Hari R. Ilmoniemi R. J. Knuutila J. Lounasmaa O. V. (1993). Magnetoencephalography—theory, instrumentation, and applications to noninvasive studies of the working human brain. Rev. Mod. Phys. 65, 413–497. doi: 10.1103/RevModPhys.65.413

[ref20] Hämäläinen M. Huang M. Bowyer S. M. (2020). Magnetoencephalography signal processing, forward modeling, inverse source imaging, and coherence analysis. Neuroimaging Clin. 30, 125–143. doi: 10.1016/j.nic.2020.02.001 32336402

[ref21] He J. Cui J. Zhang G. Xue M. Chu D. Zhao Y. (2022). Spatial–temporal seizure detection with graph attention network and bi-directional LSTM architecture. Biomed. Signal Process. Control 78:103908. doi: 10.1016/j.bspc.2022.103908

[ref22] He H. Wu D. (2020). Different set domain adaptation for brain-computer interfaces: a label alignment approach. IEEE Trans. Neural Syst. Rehabil. Eng. 28, 1091–1108. doi: 10.1109/TNSRE.2020.2980299 32167903

[ref23] Hesse C. Oostenveld R. Heskes T. Jensen O. (2007). On the development of a brain--computer interface system using high--density magnetoencephalogram signals for real--time control of a robot arm. *IEEE*.

[ref24] Ho Y. Wookey S. (2019). The real-world-weight cross-entropy loss function: modeling the costs of mislabeling. IEEE Access 8, 4806–4813. doi: 10.1109/ACCESS.2019.2962617, PMID: 39573497

[ref25] Hooda N. Kumar N. (2020). Cognitive imagery classification of EEG signals using CSP-based feature selection method. IETE Tech. Rev. 37, 315–326. doi: 10.1080/02564602.2019.1620138

[ref26] Hou Y. Jia S. Lun X. Hao Z. Shi Y. Li Y.. (2022). GCNs-net: a graph convolutional neural network approach for decoding time-resolved eeg motor imagery signals. IEEE Trans. Neural Netw. Learn. Syst. 35, 7312–7323. doi: 10.1109/TNNLS.2022.3202569, PMID: 36099220

[ref27] Hu X. Yuan S. Xu F. Leng Y. Yuan K. Yuan Q. (2020). Scalp EEG classification using deep Bi-LSTM network for seizure detection. Comput. Biol. Med. 124:103919. doi: 10.1016/j.compbiomed.2020.103919, PMID: 32771673

[ref28] Jerbi K. Lachaux J.-P. N’Diaye K. Pantazis D. Leahy R. M. Garnero L.. (2007). Coherent neural representation of hand speed in humans revealed by MEG imaging. Proc. Natl. Acad. Sci. 104, 7676–7681. doi: 10.1073/pnas.0609632104, PMID: 17442753 PMC1863498

[ref29] Ju C. Guan C. (2022). Tensor-cspnet: a novel geometric deep learning framework for motor imagery classification. IEEE Trans. Neural Netw. Learn. Syst. 34, 10955–10969. doi: 10.1109/TNNLS.2022.3172108, PMID: 35749326

[ref30] Kim H. Kim J. S. Chung C. K. (2023). Identification of cerebral cortices processing acceleration, velocity, and position during directional reaching movement with deep neural network and explainable AI. Neuro Image 266:119783. doi: 10.1016/j.neuroimage.2022.119783, PMID: 36528312

[ref31] Lahiri A. Bairagya S. Bera S. Haldar S. Biswas P. K. (2020). Lightweight modules for efficient deep learning based image restoration. IEEE Trans. Circuits Syst. Video Technol. 31, 1395–1410. doi: 10.1109/TCSVT.2020.3007723

[ref32] Lawhern V. J. Solon A. J. Waytowich N. R. Gordon S. M. Hung C. P. Lance B. J. (2018). EEGNet: a compact convolutional neural network for EEG-based brain–computer interfaces. J. Neural Eng. 15:056013. doi: 10.1088/1741-2552/aace8c, PMID: 29932424

[ref33] Leeuwis N. Paas A. Alimardani M. (2021). Vividness of visual imagery and personality impact motor-imagery brain computer interfaces. Front. Hum. Neurosci. 15:634748. doi: 10.3389/fnhum.2021.634748, PMID: 33889080 PMC8055841

[ref34] Li X. Chen J. Shi N. Yang C. Gao P. Chen X.. (2023). A hybrid steady-state visual evoked response-based brain-computer interface with MEG and EEG. Expert Syst. Appl. 223:119736. doi: 10.1016/j.eswa.2023.119736

[ref35] Lotte F. Bougrain L. Cichocki A. Clerc M. Congedo M. Rakotomamonjy A.. (2018). A review of classification algorithms for EEG-based brain–computer interfaces: a 10 year update. J. Neural Eng. 15:031005. doi: 10.1088/1741-2552/aab2f2, PMID: 29488902

[ref36] Lu H. Eng H.-L. Guan C. Plataniotis K. N. Venetsanopoulos A. N. (2010). Regularized common spatial pattern with aggregation for EEG classification in small-sample setting. IEEE Trans. Biomed. Eng. 57, 2936–2946. doi: 10.1109/TBME.2010.2082540, PMID: 20889425

[ref37] Luo T.-J. Zhou C.-L. Chao F. (2018). Exploring spatial-frequency-sequential relationships for motor imagery classification with recurrent neural network. BMC Bioinformatics 19, 1–18. doi: 10.1186/s12859-018-2365-1 30268089 PMC6162908

[ref38] Ma J. Ma W. Zhang J. Li Y. Yang B. Shan C. (2024). Partial prior transfer learning based on self-attention CNN for EEG decoding in stroke patients. Sci. Rep. 14:28170. doi: 10.1038/s41598-024-79202-8 39548177 PMC11568294

[ref39] Ma W. Wang C. Sun X. Lin X. Wang Y. (2023). A double-branch graph convolutional network based on individual differences weakening for motor imagery EEG classification. Biomed. Signal Process. Control 84:104684. doi: 10.1016/j.bspc.2023.104684

[ref40] Mane R. Chew E. Chua K. Ang K. K. Robinson N. Vinod A. P.. (2021). FBCNet: a multi-view convolutional neural network for brain-computer interface. arXiv [Preprint]. doi: 10.48550/arXiv.2104.01233

[ref41] Mane R. Chouhan T. Guan C. (2020). BCI for stroke rehabilitation: motor and beyond. J. Neural Eng. 17:041001. doi: 10.1088/1741-2552/aba162, PMID: 32613947

[ref42] Mane R. Robinson N. Vinod A. P. Lee S.-W. Guan C. (2020). A multi-view CNN with novel variance layer for motor imagery brain computer interface. Proceedings of the 2020 42nd annual international conference of the IEEE engineering in medicine & biology society (EMBC). IEEE. 2950–2953. 10.1109/EMBC44109.2020.9175874 33018625

[ref43] Mellinger J. Schalk G. Braun C. Preissl H. Rosenstiel W. Birbaumer N.. (2007). An MEG-based brain–computer interface (BCI). NeuroImage 36, 581–593. doi: 10.1016/j.neuroimage.2007.03.019, PMID: 17475511 PMC2017111

[ref44] Nara S. Raza H. Carreiras M. Molinaro N. (2023). Decoding numeracy and literacy in the human brain: insights from MEG and MVPA. Sci. Rep. 13:10979. doi: 10.1038/s41598-023-37113-0, PMID: 37414784 PMC10326015

[ref45] Peng H. Gong W. Beckmann C. F. Vedaldi A. Smith S. M. (2021). Accurate brain age prediction with lightweight deep neural networks. Med. Image Anal. 68:101871. doi: 10.1016/j.media.2020.101871, PMID: 33197716 PMC7610710

[ref46] Phadikar S. Sinha N. Ghosh R. (2023). Unsupervised feature extraction with autoencoders for EEG based multiclass motor imagery BCI. Expert Syst. Appl. 213:118901. doi: 10.1016/j.eswa.2022.118901

[ref47] Piqueira J. R. C. (2011). Network of phase-locking oscillators and a possible model for neural synchronization. Commun. Nonlinear Sci. Numer. Simul. 16, 3844–3854. doi: 10.1016/j.cnsns.2010.12.031

[ref48] Ramoser H. Muller-Gerking J. Pfurtscheller G. (2000). Optimal spatial filtering of single trial EEG during imagined hand movement. IEEE Trans. Rehabil. Eng. 8, 441–446. doi: 10.1109/86.895946, PMID: 11204034

[ref49] Rathee D. Raza H. Roy S. Prasad G. (2021). A magnetoencephalography dataset for motor and cognitive imagery-based brain-computer interface. Sci. Data 8:120. doi: 10.1038/s41597-021-00899-7, PMID: 33927204 PMC8085139

[ref50] Roy Y. Banville H. Albuquerque I. Gramfort A. Falk T. H. Faubert J. (2019). Deep learning-based electroencephalography analysis: a systematic review. J. Neural Eng. 16:051001. doi: 10.1088/1741-2552/ab260c, PMID: 31151119

[ref51] Saichand N. V. (2021). Epileptic seizure detection using novel multilayer LSTM discriminant network and dynamic mode Koopman decomposition. Biomed. Signal Process. Control 68:102723. doi: 10.1016/j.bspc.2021.102723

[ref52] Sakhavi S. Guan C. Yan S. (2018). Learning temporal information for brain-computer interface using convolutional neural networks. IEEE Trans. Neural Netw. Learn. Syst. 29, 5619–5629. doi: 10.1109/TNNLS.2018.2789927, PMID: 29994075

[ref53] Sakhavi S. Guan C. Yan S. (2015). Parallel convolutional-linear neural network for motor imagery classification. Proceedings of the 2015 23rd European signal processing conference (EUSIPCO), Nice, France. 2736–2740.

[ref54] Sarma M. Bond C. Nara S. Raza H. (2023). MEGNet: a MEG-based deep learning model for cognitive and motor imagery classification. Proceedings of the 2023 IEEE international conference on bioinformatics and biomedicine (BIBM), Istanbul, Turkiye. 2571–2578.

[ref55] Schirrmeister R. T. Springenberg J. T. Fiederer L. D. J. Glasstetter M. Eggensperger K. Tangermann M.. (2017). Deep learning with convolutional neural networks for EEG decoding and visualization. Hum. Brain Mapp. 38, 5391–5420. doi: 10.1002/hbm.23730, PMID: 28782865 PMC5655781

[ref56] Solodkin A. Hlustik P. Chen E. E. Small S. L. (2004). Fine modulation in network activation during motor execution and motor imagery. Cereb. Cortex 14, 1246–1255. doi: 10.1093/cercor/bhh086, PMID: 15166100

[ref57] Song T. Zheng W. Song P. Cui Z. (2018). EEG emotion recognition using dynamical graph convolutional neural networks. IEEE Trans. Affect. Comput. 11, 532–541. doi: 10.1109/TAFFC.2018.2817622, PMID: 39573497

[ref58] Tang C. Gao T. Wang G. Chen B. (2024). Coherence-based channel selection and Riemannian geometry features for magnetoencephalography decoding. Cogn. Neurodyn. 18, 3535–3548. doi: 10.1007/s11571-024-10085-1, PMID: 39712116 PMC11655792

[ref59] Thomas K. P. Guan C. Lau C. T. Vinod A. P. Ang K. K. (2009). A new discriminative common spatial pattern method for motor imagery brain–computer interfaces. IEEE Trans. Biomed. Eng. 56, 2730–2733. doi: 10.1109/TBME.2009.2026181, PMID: 19605314

[ref60] Van Der Maaten L. Hinton G. (2008). Visualizing data using t-SNE. J. Mach. Learn. Res. 9, 2579–2605.

[ref61] Wang P. Jiang A. Liu X. Shang J. Zhang L. (2018). LSTM-based EEG classification in motor imagery tasks. IEEE Trans. Neural Syst. Rehabil. Eng. 26, 2086–2095. doi: 10.1109/TNSRE.2018.2876129, PMID: 30334800

[ref62] Wang Z. Tong Y. Heng X. (2019). Phase-locking value based graph convolutional neural networks for emotion recognition. IEEE Access 7, 93711–93722. doi: 10.1109/ACCESS.2019.2927768

[ref63] Wang X. Zheng Y. Wang F. Ding H. Meng J. Zhuo Y. (2024). Unilateral movement decoding of upper and lower limbs using magnetoencephalography. Biomed. Signal Process. Control 93:106215. doi: 10.1016/j.bspc.2024.106215

[ref64] Yang J. Huang X. Wu H. Yang X. (2020). EEG-based emotion classification based on bidirectional long short-term memory network. Proc. Comput. Sci. 174, 491–504. doi: 10.1016/j.procs.2020.06.117

[ref65] Yang Y. Luo S. Wang W. Gao X. Yao X. Wu T. (2024). From bench to bedside: overview of magnetoencephalography in basic principle, signal processing, source localization and clinical applications. Neuro Image Clin. 42:103608. doi: 10.1016/j.nicl.2024.103608, PMID: 38653131 PMC11059345

[ref66] Youssofzadeh V. Roy S. Chowdhury A. Izadysadr A. Parkkonen L. Raghavan M.. (2023). Mapping and decoding cortical engagement during motor imagery, mental arithmetic, and silent word generation using MEG. Hum. Brain Mapp. 44, 3324–3342. doi: 10.1002/hbm.26284, PMID: 36987698 PMC10171552

[ref67] Yu Y. Si X. Hu C. Zhang J. (2019). A review of recurrent neural networks: LSTM cells and network architectures. Neural Comput. 31, 1235–1270. doi: 10.1162/neco_a_01199, PMID: 31113301

[ref68] Zhang K. Robinson N. Lee S.-W. Guan C. (2021). Adaptive transfer learning for EEG motor imagery classification with deep convolutional neural network. Neural Netw. 136, 1–10. doi: 10.1016/j.neunet.2020.12.013, PMID: 33401114

[ref69] Zhao L. Song Y. Zhang C. Liu Y. Wang P. Lin T.. (2019). T-GCN: a temporal graph convolutional network for traffic prediction. IEEE Trans. Intell. Transp. Syst. 21, 3848–3858. doi: 10.1109/TITS.2019.2935152

[ref70] Zheng Q. Zhu F. Heng P.-A. (2018). Robust support matrix machine for single trial EEG classification. IEEE Trans. Neural Syst. Rehabil. Eng. 26, 551–562. doi: 10.1109/TNSRE.2018.2794534, PMID: 29522399

