# Supplementary material for: Recognition of brain activities via graph-based long short-term memory-convolutional neural network
Source: Front Neurosci. 2025 Mar 24;19:1546559. doi: 10.3389/fnins.2025.1546559 (PMC11973346; doi:10.3389/fnins.2025.1546559)
Supplement: Supplementary file 1 [file Data_Sheet_1.pdf]

Supplementary Materials

Recognition of brain activities via graph-based long short-term memory-convolutional neural network

Yanling Yang, Helong Zhao, Zezhou Hao, Cheng Shi, Liang Zhou, Xufeng Yao*

Suppl. Table 1

Table S1 The Classification accuracy, kappa and F1-score for Hand VS. Feet (H-F) classification

Sub.	FBCSP			FBCNet			EEGNet			Deep ConvNets			Shallow ConvNet			MEGNet			GLCNet		
	Acc(%)	K	F1	Acc(%)	K	F1	Acc(%)	K	F1	Acc(%)	K	F1	Acc(%)	K	F1	Acc(%)	K	F1	Acc(%)	K	F1
1	61.0	0.22	0.60	56.0	0.16	0.49	58.0	0.14	0.57	55.0	0.14	0.57	53.0	0.10	0.55	65.0	0.30	0.65	61.0	0.20	0.56
3	71.0	0.42	0.70	79.0	0.60	0.80	67.0	0.30	0.65	68.0	0.30	0.65	69.0	0.20	0.60	69.0	0.38	0.69	83.0	0.62	0.81
4	55.0	0.10	0.46	50.0	0.02	0.44	56.0	0.06	0.53	42.0	0.06	0.53	57.0	0.14	0.57	50.0	0.01	0.42	64.0	0.26	0.63
6	53.0	0.06	0.41	75.0	0.44	0.72	81.0	0.56	0.78	81.0	0.56	0.78	58.0	0.18	0.57	80.0	0.60	0.80	80.0	0.60	0.80
7	60.0	0.20	0.54	53.0	0.04	0.41	63.0	0.12	0.55	54.0	0.12	0.55	46.0	0.00	0.50	59.0	0.18	0.59	57.0	0.14	0.52
9	54.0	0.08	0.46	55.0	0.16	0.53	63.0	0.30	0.65	62.0	0.30	0.65	76.0	0.48	0.74	61.0	0.22	0.60	71.0	0.30	0.65
11	53.0	0.06	0.44	56.0	0.22	0.59	63.0	0.26	0.63	56.0	0.26	0.63	52.0	0.14	0.56	60.0	0.20	0.55	75.0	0.40	0.70
12	56.0	0.12	0.51	61.0	0.24	0.60	54.0	0.00	0.50	54.0	0.00	0.50	62.0	0.24	0.60	54.0	0.08	0.54	60.0	0.20	0.59
13	57.0	0.14	0.50	66.0	0.36	0.67	72.0	0.40	0.69	55.0	0.40	0.69	55.0	0.08	0.54	61.0	0.22	0.61	73.0	0.46	0.73
14	67.0	0.34	0.67	71.0	0.28	0.64	56.0	0.16	0.58	57.0	0.16	0.58	55.0	0.14	0.57	57.0	0.14	0.57	75.0	0.48	0.74
15	65.0	0.30	0.62	85.0	0.68	0.84	72.0	0.40	0.70	65.0	0.40	0.70	76.0	0.46	0.72	75.0	0.50	0.75	87.0	0.72	0.86
16	54.0	0.08	0.42	58.0	0.10	0.55	57.0	0.08	0.42	60.0	0.08	0.42	45.0	0.16	0.42	63.0	0.26	0.59	59.0	0.16	0.57
17	56.0	0.12	0.52	51.0	0.06	0.52	56.0	0.08	0.54	68.0	0.08	0.54	55.0	0.06	0.49	60.0	0.20	0.60	62.0	0.26	0.60
18	67.0	0.34	0.66	77.0	0.54	0.77	79.0	0.54	0.77	67.0	0.54	0.77	52.0	0.00	0.50	81.0	0.62	0.81	75.0	0.48	0.74
19	52.0	0.04	0.38	70.0	0.34	0.67	81.0	0.62	0.81	56.0	0.62	0.81	63.0	0.16	0.57	75.0	0.50	0.75	76.0	0.32	0.64
20	64.0	0.28	0.62	74.0	0.46	0.72	70.0	0.44	0.71	54.0	0.44	0.71	64.0	0.16	0.53	73.0	0.46	0.73	75.0	0.46	0.73
Avg.	59.1	0.20	0.50	64.9	0.29	0.62	65.5	0.28	0.63	59.6	0.28	0.63	58.6	0.17	0.56	65.2	0.30	0.64	**70.8**	**0.38**	**0.68**

Suppl. Table 2

Table S2 The Classification accuracy, kappa and F1-score for Hand VS. Word (H-W) classification

Sub.	FBCSP			FBCNet			EEGNet			Deep ConvNets			Shallow ConvNet			MEGNet			GLCNet		
	Acc(%)	K	F1	Acc(%)	K	F1	Acc(%)	K	F1	Acc(%)	K	F1	Acc(%)	K	F1	Acc(%)	K	F1	Acc(%)	K	F1
1	53.0	0.06	0.40	76.0	0.58	0.78	71.0	0.34	0.66	68.0	0.34	0.66	51.0	0.02	0.37	70.0	0.40	0.70	80.0	0.58	0.79
3	92.0	0.84	0.92	97.0	0.88	0.94	78.0	0.48	0.74	73.0	0.48	0.74	96.0	0.92	0.96	76.0	0.52	0.76	98.0	0.96	0.98
4	52.0	0.04	0.51	50.0	0.02	0.43	51.0	0.04	0.52	52.0	0.04	0.52	52.0	0.12	0.52	53.0	0.06	0.53	61.0	0.26	0.63
6	52.0	0.04	0.38	86.0	0.44	0.70	84.0	0.68	0.84	80.0	0.68	0.84	62.0	0.22	0.55	82.0	0.64	0.82	86.0	0.76	0.88
7	83.0	0.66	0.83	55.0	0.10	0.47	64.0	0.20	0.60	59.0	0.20	0.60	55.0	0.10	0.50	70.0	0.40	0.68	76.0	0.52	0.76
9	54.0	0.08	0.42	84.0	0.66	0.83	73.0	0.42	0.71	72.0	0.42	0.71	65.0	0.42	0.69	76.0	0.52	0.75	86.0	0.78	0.89
11	60.0	0.20	0.52	84.0	0.68	0.84	75.0	0.48	0.73	75.0	0.48	0.73	81.0	0.60	0.80	71.0	0.42	0.71	86.0	0.68	0.84
12	53.0	0.06	0.40	81.0	0.58	0.78	75.0	0.46	0.72	63.0	0.46	0.72	69.0	0.30	0.63	67.0	0.34	0.67	84.0	0.70	0.85
13	82.0	0.64	0.82	89.0	0.72	0.86	73.0	0.44	0.72	71.0	0.44	0.72	71.0	0.40	0.70	77.0	0.54	0.77	85.0	0.70	0.85
14	61.0	0.22	0.54	81.0	0.54	0.77	68.0	0.40	0.70	62.0	0.40	0.70	69.0	0.30	0.64	68.0	0.36	0.68	82.0	0.60	0.79
15	73.0	0.46	0.71	90.0	0.76	0.88	80.0	0.62	0.81	78.0	0.62	0.81	72.0	0.44	0.72	83.0	0.66	0.83	93.0	0.90	0.95
16	54.0	0.08	0.42	86.0	0.70	0.85	82.0	0.44	0.72	74.0	0.44	0.72	80.0	0.52	0.76	75.0	0.50	0.75	84.0	0.66	0.83
17	65.0	0.30	0.65	56.0	0.16	0.52	58.0	0.12	0.56	58.0	0.12	0.56	65.0	0.28	0.64	66.0	0.32	0.66	60.0	0.28	0.64
18	84.0	0.68	0.84	86.0	0.62	0.81	77.0	0.52	0.76	84.0	0.52	0.76	71.0	0.34	0.67	76.0	0.52	0.76	90.0	0.80	0.90
19	55.0	0.10	0.47	80.0	0.58	0.79	69.0	0.36	0.68	67.0	0.36	0.68	74.0	0.40	0.70	67.0	0.34	0.69	79.0	0.56	0.78
20	87.0	0.74	0.87	92.0	0.84	0.92	80.0	0.60	0.80	84.0	0.60	0.80	90.0	0.72	0.86	75.0	0.50	0.75	93.0	0.88	0.94
Avg.	66.3	0.33	0.60	79.6	0.55	0.76	72.4	0.41	0.70	70.0	0.41	0.70	70.2	0.38	0.67	72.0	0.44	0.72	**82.7**	**0.66**	**0.83**

Suppl. Table 3

Table S3 The Classification accuracy, kappa and F1-score for Hand VS. Sub (H-S) classification

Sub.	FBCSP			FBCNet			EEGNet			Deep ConvNets			Shallow ConvNet			MEGNet			GLCNet		
	Acc(%)	K	F1	Acc(%)	K	F1	Acc(%)	K	F1	Acc(%)	K	F1	Acc(%)	K	F1	Acc(%)	K	F1	Acc(%)	K	F1
1	59.0	0.18	0.52	75.0	0.56	0.78	79.0	0.56	0.78	65.0	0.56	0.78	47.0	0.04	0.34	76.0	0.52	0.76	74.0	0.48	0.74
3	90.0	0.80	0.90	98.0	0.92	0.96	87.0	0.76	0.88	88.0	0.76	0.88	94.0	0.82	0.91	78.0	0.56	0.78	97.0	0.92	0.96
4	55.0	0.10	0.50	50.0	0.00	0.35	58.0	0.18	0.58	57.0	0.18	0.58	51.0	0.04	0.47	54.0	0.08	0.52	68.0	0.28	0.64
6	55.0	0.10	0.44	91.0	0.82	0.91	90.0	0.78	0.89	93.0	0.78	0.89	78.0	0.52	0.76	88.0	0.76	0.88	95.0	0.88	0.94
7	51.0	0.02	0.36	53.0	0.04	0.39	78.0	0.60	0.80	71.0	0.60	0.80	47.0	0.08	0.39	75.0	0.50	0.74	58.0	0.18	0.53
9	71.0	0.42	0.68	73.0	0.42	0.69	85.0	0.64	0.82	67.0	0.64	0.82	84.0	0.70	0.85	71.0	0.42	0.70	91.0	0.88	0.94
11	92.0	0.84	0.92	96.0	0.90	0.95	82.0	0.64	0.82	89.0	0.64	0.82	92.0	0.86	0.93	81.0	0.62	0.81	94.0	0.88	0.94
12	55.0	0.10	0.44	75.0	0.50	0.74	79.0	0.46	0.73	63.0	0.46	0.73	70.0	0.44	0.71	67.0	0.34	0.66	81.0	0.64	0.82
13	54.0	0.08	0.42	83.0	0.64	0.82	77.0	0.52	0.76	65.0	0.52	0.76	77.0	0.60	0.80	75.0	0.50	0.75	84.0	0.64	0.82
14	61.0	0.22	0.61	71.0	0.26	0.58	68.0	0.26	0.63	69.0	0.26	0.63	62.0	0.22	0.61	68.0	0.36	0.68	70.0	0.42	0.70
15	54.0	0.08	0.52	85.0	0.62	0.81	83.0	0.68	0.84	81.0	0.68	0.84	87.0	0.74	0.87	87.0	0.74	0.87	90.0	0.82	0.91
16	58.0	0.16	0.50	91.0	0.74	0.87	84.0	0.62	0.81	80.0	0.62	0.81	80.0	0.60	0.79	91.0	0.82	0.91	81.0	0.60	0.80
17	52.0	0.04	0.44	74.0	0.36	0.68	76.0	0.44	0.72	72.0	0.44	0.72	66.0	0.24	0.62	70.0	0.40	0.70	73.0	0.54	0.77
18	95.0	0.90	0.95	91.0	0.82	0.91	93.0	0.84	0.92	89.0	0.84	0.92	78.0	0.46	0.73	88.0	0.76	0.88	90.0	0.78	0.89
19	51.0	0.02	0.51	69.0	0.44	0.72	79.0	0.50	0.75	81.0	0.50	0.75	57.0	0.12	0.55	76.0	0.52	0.76	63.0	0.28	0.64
20	95.0	0.90	0.95	89.0	0.82	0.91	84.0	0.68	0.84	60.0	0.68	0.84	84.0	0.66	0.83	86.0	0.72	0.86	92.0	0.88	0.94
Avg.	65.5	0.31	0.60	79.0	0.55	0.75	80.1	0.57	0.78	74.4	0.57	0.78	72.1	0.45	0.70	76.9	0.54	0.77	**81.3**	**0.63**	**0.81**

Suppl. Table 4

Table S4 The Classification accuracy, kappa and F1-score for Feet VS. Word (F-W) classification

Sub.	FBCSP			FBCNet			EEGNet			Deep ConvNets			Shallow ConvNet			MEGNet			GLCNet		
	Acc(%)	K	F1	Acc(%)	K	F1	Acc(%)	K	F1	Acc(%)	K	F1	Acc(%)	K	F1	Acc(%)	K	F1	Acc(%)	K	F1
1	62.0	0.24	0.57	81.0	0.58	0.79	71.0	0.46	0.73	63.0	0.46	0.73	59.0	0.10	0.51	63.0	0.26	0.63	65.0	0.34	0.63
3	57.0	0.14	0.48	92.0	0.84	0.92	81.0	0.62	0.81	81.0	0.62	0.81	93.0	0.86	0.93	80.0	0.60	0.80	94.0	0.86	0.93
4	50.0	0.00	0.33	52.0	0.08	0.47	59.0	0.16	0.58	63.0	0.16	0.58	60.0	0.22	0.61	57.0	0.14	0.57	69.0	0.34	0.65
6	54.0	0.08	0.42	83.0	0.68	0.84	80.0	0.54	0.77	82.0	0.54	0.77	60.0	0.22	0.58	85.0	0.70	0.85	70.0	0.36	0.66
7	73.0	0.46	0.72	63.0	0.24	0.57	66.0	0.32	0.66	59.0	0.32	0.66	72.0	0.30	0.65	65.0	0.30	0.65	70.0	0.38	0.68
9	56.0	0.12	0.54	82.0	0.62	0.80	81.0	0.50	0.74	77.0	0.50	0.74	63.0	0.18	0.56	74.0	0.48	0.74	85.0	0.60	0.80
11	59.0	0.18	0.51	89.0	0.74	0.87	75.0	0.50	0.74	80.0	0.50	0.74	86.0	0.70	0.85	84.0	0.68	0.84	86.0	0.72	0.86
12	54.0	0.08	0.44	68.0	0.36	0.65	72.0	0.48	0.74	62.0	0.48	0.74	70.0	0.40	0.67	73.0	0.46	0.73	73.0	0.46	0.72
13	74.0	0.48	0.72	83.0	0.62	0.81	70.0	0.40	0.70	61.0	0.40	0.70	80.0	0.50	0.75	71.0	0.42	0.71	77.0	0.52	0.76
14	57.0	0.14	0.51	74.0	0.24	0.60	70.0	0.46	0.73	55.0	0.46	0.73	59.0	0.14	0.57	69.0	0.38	0.69	57.0	0.14	0.47
15	63.0	0.26	0.63	86.0	0.72	0.86	77.0	0.50	0.75	66.0	0.50	0.75	81.0	0.62	0.81	73.0	0.46	0.73	83.0	0.62	0.80
16	72.0	0.44	0.70	89.0	0.70	0.85	69.0	0.02	0.36	72.0	0.02	0.36	57.0	0.02	0.48	54.0	0.08	0.43	83.0	0.68	0.84
17	58.0	0.16	0.56	51.0	0.02	0.39	66.0	0.32	0.66	62.0	0.32	0.66	57.0	0.12	0.56	63.0	0.26	0.61	63.0	0.26	0.61
18	62.0	0.24	0.61	76.0	0.52	0.76	80.0	0.54	0.77	65.0	0.54	0.77	80.0	0.56	0.78	74.0	0.48	0.74	77.0	0.50	0.75
19	59.0	0.18	0.57	83.0	0.64	0.82	74.0	0.58	0.79	76.0	0.58	0.79	74.0	0.48	0.74	81.0.	0.62	0.81	85.0	0.70	0.85
20	72.0	0.44	0.70	94.0	0.88	0.94	82.0	0.48	0.74	72.0	0.48	0.74	83.0	0.68	0.84	80.0	0.60	0.80	87.0	0.78	0.89
Avg.	61.4	0.20	0.60	**77.9**	**0.53**	**0.75**	73.3	0.43	0.70	68.5	0.43	0.70	70.8	0.38	0.68	71.6	0.42	0.70	76.5	0.52	0.74

Suppl. Table 5

Table S5 The Classification accuracy, kappa and F1-score for Feet VS. Sub (F-S) classification

Sub.	FBCSP			FBCNet			EEGNet			Deep ConvNets			Shallow ConvNet			MEGNet			GLCNet		
	Acc(%)	K	F1	Acc(%)	K	F1	Acc(%)	K	F1	Acc(%)	K	F1	Acc(%)	K	F1	Acc(%)	K	F1	Acc(%)	K	F1
1	79.0	0.58	0.79	66.0	0.28	0.60	75.0	0.52	0.76	63.0	0.52	0.76	47.0	0.01	0.42	50.0	0.08	0.30	72.0	0.48	0.73
3	71.0	0.42	0.71	94.0	0.86	0.93	84.0	0.68	0.84	87.0	0.68	0.84	89.0	0.76	0.88	55.0	0.09	0.36	93.0	0.86	0.93
4	63.0	0.26	0.63	52.0	0.04	0.40	60.0	0.24	0.62	50.0	0.24	0.62	55.0	0.08	0.54	50.0	0.08	0.30	70.0	0.40	0.70
6	65.0	0.30	0.65	94.0	0.86	0.93	92.0	0.78	0.89	87.0	0.78	0.89	85.0	0.64	0.82	60.0	0.09	0.39	93.0	0.72	0.86
7	69.0	0.38	0.69	55.0	0.08	0.43	74.0	0.64	0.82	84.0	0.64	0.82	61.0	0.16	0.55	25.0	0.04	0.20	72.0	0.48	0.73
9	68.0	0.36	0.68	90.0	0.78	0.89	79.0	0.58	0.79	88.0	0.58	0.79	85.0	0.70	0.85	50.0	0.08	0.30	89.0	0.80	0.90
11	93.0	0.86	0.93	89.0	0.78	0.89	50.0	0.00	0.33	80.0	0.00	0.33	87.0	0.68	0.84	40.0	0.05	0.28	92.0	0.84	0.92
12	61.0	0.22	0.55	62.0	0.24	0.57	63.0	0.36	0.68	59.0	0.36	0.68	65.0	0.30	0.63	40.0	0.05	0.28	83.0	0.66	0.83
13	88.0	0.76	0.88	83.0	0.64	0.82	75.0	0.52	0.76	69.0	0.52	0.76	79.0	0.62	0.81	60.0	0.06	0.39	85.0	0.68	0.84
14	72.0	0.44	0.70	67.0	0.34	0.65	69.0	0.32	0.66	69.0	0.32	0.66	66.0	0.28	0.64	65.0	0.07	0.39	65.0	0.28	0.59
15	62.0	0.24	0.60	91.0	0.80	0.90	75.0	0.48	0.74	67.0	0.48	0.74	86.0	0.70	0.85	60.0	0.06	0.39	93.0	0.82	0.91
16	69.0	0.38	0.68	90.0	0.66	0.83	90.0	0.76	0.88	81.0	0.76	0.88	74.0	0.46	0.72	40.0	0005	0.28	92.0	0.76	0.88
17	45.0	0.10	0.45	57.0	0.16	0.51	64.0	0.32	0.66	61.0	0.32	0.66	58.0	0.12	0.55	55.0	0.09	0.36	62.0	0.22	0.61
18	68.0	0.36	0.68	74.0	0.46	0.73	78.0	0.56	0.78	70.0	0.56	0.78	77.0	0.48	0.74	50.0	0.08	0.30	83.0	0.70	0.85
19	55.0	0.10	0.46	78.0	0.52	0.76	77.0	0.58	0.79	75.0	0.58	0.79	56.0	0.10	0.55	55.0	0.09	0.36	72.0	0.50	0.75
20	89.0	0.78	0.89	90.0	0.70	0.85	79.0	0.58	0.79	78.0	0.58	0.79	91.0	0.78	0.89	50.0	0.08	0.30	91.0	0.78	0.89
Avg.	69.8	0.40	0.70	77.0	0.51	0.73	74.0	0.50	0.74	73.0	0.50	0.74	72.6	0.43	0.70	50.3	0.07	0.32	**81.7**	**0.62**	**0.81**

Suppl. Table 6

Table S6 The Classification accuracy, kappa and F1-score for Word VS. Sub (W-S) classification

Sub.	FBCSP			FBCNet			EEGNet			Deep ConvNets			Shallow ConvNet			MEGNet			GLCNet		
	Acc(%)	K	F1	Acc(%)	K	F1	Acc(%)	K	F1	Acc(%)	K	F1	Acc(%)	K	F1	Acc(%)	K	F1	Acc(%)	K	F1
1	52.0	0.04	0.38	63.0	0.16	0.50	74.0	0.32	0.66	52.0	0.32	0.66	55.0	0.01	0.42	73.0	0.46	0.73	74.0	0.42	0.71
3	53.0	0.06	0.40	94.0	0.84	0.92	74.0	0.50	0.75	81.0	0.50	0.75	82.0	0.50	0.75	75.0	0.50	0.74	83.0	0.68	0.84
4	56.0	0.12	0.47	49.0	0.02	0.46	53.0	0.08	0.54	52.0	0.08	0.54	49.0	0.04	0.52	53.0	0.06	0.46	59.0	0.22	0.61
6	52.0	0.04	0.38	90.0	0.80	0.90	77.0	0.46	0.73	81.0	0.46	0.73	57.0	0.18	0.55	75.0	0.50	0.74	89.0	0.68	0.84
7	54.0	0.08	0.43	53.0	0.22	0.58	59.0	0.20	0.60	70.0	0.20	0.60	57.0	0.12	0.55	73.0	0.46	0.73	56.0	0.12	0.45
9	54.0	0.08	0.42	82.0	0.54	0.76	79.0	0.60	0.80	86.0	0.60	0.80	51.0	0.02	0.36	84.0	0.68	0.84	87.0	0.80	0.90
11	84.0	0.68	0.84	94.0	0.88	0.94	70.0	0.00	0.33	66.0	0.00	0.33	91.0	0.86	0.93	50.0	0.01	0.33	95.0	0.90	0.95
12	52.0	0.04	0.38	74.0	0.46	0.73	69.0	0.36	0.68	53.0	0.36	0.68	55.0	0.10	0.52	61.0	0.22	0.61	76.0	0.54	0.76
13	53.0	0.06	0.40	74.0	0.50	0.75	69.0	0.38	0.69	71.0	0.38	0.69	62.0	0.18	0.59	73.0	0.46	0.73	78.0	0.56	0.78
14	52.0	0.04	0.38	77.0	0.50	0.74	67.0	0.22	0.61	71.0	0.22	0.61	55.0	0.10	0.55	69.0	0.38	0.69	78.0	0.58	0.79
15	62.0	0.24	0.60	82.0	0.66	0.83	77.0	0.60	0.80	78.0	0.60	0.80	75.0	0.48	0.74	74.0	0.48	0.74	90.0	0.78	0.89
16	53.0	0.06	0.40	86.0	0.78	0.89	78.0	0.44	0.72	71.0	0.44	0.72	80.0	0.60	0.80	68.0	0.36	0.68	81.0	0.66	0.83
17	51.0	0.02	0.47	68.0	0.38	0.68	66.0	0.22	0.61	59.0	0.22	0.61	61.0	0.22	0.60	73.0	0.46	0.73	63.0	0.30	0.62
18	55.0	0.10	0.44	86.0	0.72	0.86	89.0	0.76	0.88	81.0	0.76	0.88	73.0	0.40	0.70	84.0	0.68	0.84	86.0	0.74	0.87
19	73.0	0.46	0.72	71.0	0.42	0.69	81.0	0.62	0.81	77.0	0.62	0.81	80.0	0.54	0.77	77.0	0.54	0.77	80.0	0.56	0.78
20	90.0	0.80	0.90	80.0	0.46	0.72	79.0	0.56	0.78	73.0	0.56	0.78	77.0	0.54	0.77	81.0	0.62	0.81	86.0	0.72	0.86
Avg.	59.1	0.18	0.50	76.4	0.52	0.75	72.6	0.40	0.69	70.0	0.40	0.69	66.3	0.31	0.63	71.4	0.43	0.70	**78.9**	**0.58**	**0.78**
